# Cas5 Regulates the Exposure of β-Glucan, the Cell Surface Hydrophobicity, and the Expression of Cell Wall Proteins to Remodel the *Candida albicans* Cell Wall and Participates in the Recruitment of Neutrophils

**DOI:** 10.3390/microorganisms13030683

**Published:** 2025-03-19

**Authors:** Qiyue Zhang, Guanglin Li, Yanmei Wang, Chen Yang, Wenhui Bai, Qingqing Li, Jiye Zhang, Peipei Zhang

**Affiliations:** 1 School of Pharmacy, Health Science Center, Xi’an Jiaotong University, Xi’an 710061, China; qiyzhang0829@163.com (Q.Z.); liguanglin0906@163.com (G.L.); wangyanmei0329@163.com (Y.W.); xjt_yc@stu.xjtu.edu.cn (C.Y.); bwh20010202@163.com (W.B.); liqingqing0217@xjtu.edu.cn (Q.L.); zjy2011@mail.xjtu.edu.cn (J.Z.); 2 Institute of Pharmaceutical Science and Technology, Xi’an Jiaotong University, Xi’an 710061, China

**Keywords:** *Candida albicans*, Cas5, virulence, cell wall

## Abstract

*Candida albicans* (*C. albicans*) is a major opportunistic fungal pathogen that causes life-threatening infections, particularly in immunocompromised individuals, underscoring the critical need to understand its pathogenic mechanisms. This study investigates the role of Cas5, a key transcription factor, in regulating *C. albicans* cell wall remodeling, virulence, and host interactions. Genetic manipulation and biochemical assays were used to examine the effects of Cas5 depletion on *C. albicans* cell wall structure, adhesion to host cells, morphology transition, innate immune cells recruitment, and pathogenicity in a BALB/C mouse model of oropharyngeal candidiasis (OPC). The results showed that the Cas5 depletion mediated β-glucan exposure and enhanced *C. albicans*’s ability to recruit neutrophils in vivo. Additionally, Cas5-mediated changes in cell surface hydrophobicity (CSH), CWP expressions, and morphological transition promoted *C. albicans* adhesion to biologically active surfaces (host cells) and increased fungal burden in the mouse model of OPC. In conclusion, Cas5 modulates *C. albicans* cell wall remodeling by masking cell wall β-glucan, altering CSH, and regulating the expression of cell wall proteins (CWPs). Additionally, Cas5 participates in inhibiting neutrophil recruitment and enhancing the *C. albicans* adhesion to host cells, as well as facilitating morphological transitions. These actions promote the colonization and invasion of *C. albicans* in OPC pathogenesis.

## 1. Introduction

The opportunistic fungal pathogen *C. albicans* asymptomatically colonizes the oral cavity, gastrointestinal tract, and genital tract of most healthy humans but causes life-threatening infections, with a mortality rate of more than 40% in immunocompromised individuals [1,2,3,4]. Only four classes of antifungal drugs are currently approved for the treatment of disseminated candidiasis: azoles, echinocandins, polyenes, and pyrimidines [4,5]. Unfortunately, the increasing rate of antifungal resistance severely limits the effects of clinical intervention on *C. albicans* infection-related outcomes [1,6]. To fight antifungal drug-resistant *C. albicans*, the discovery of new targets is highly desirable, and only innovative drugs with new modes of action that can escape pre-existing resistance mechanisms could represent a valid solution to counteract the continuing emergence and spread of resistant infections [7].

The cell wall is the initial site of interaction between *C. albicans* and its host cells and maintains the integrity of fungal cells under environmental stresses [8,9,10]. The *C. albicans* cell wall is a bilayered structure consisting of polysaccharides and proteins [8,9,11]. The inner layer is composed of β-glucan and chitin, which act as the cell skeleton, maintaining the cell shape and rigidity [9,11,12]. In addition to its role in cellular structure, β-glucan, a pathogen-associated molecular pattern (PAMP), can be recognized by host cell pattern recognition receptors (PRRs) located at the phagocyte surface [13]. However, in general, β-glucan is largely shielded by highly branched *N*- and/or *O*-linked mannan fibrils located in the outer layer of the *C. albicans* yeast cell wall, preventing the initiation of phagocytic uptake [13,14]. In fact, fibrillar mannan is also an essential component of the *C. albicans* cell wall and plays a crucial role in host–pathogen interactions, such as the adhesion of host cells and invasion of tissues, in addition to being involved in innate immunity [15]. Cell wall proteins (CWPs) are another main component of the outer cell wall that attaches to the cell wall mannan and arranges perpendicularly to the inner layer [9,12,16]. Several CWPs have been found to be associated with various virulence factors in *C. albicans* [16,17,18]. For example, Hwp1 is required for normal biofilm formation both on cardiovascular instrument silicone sheets and in a rat catheter infection model [19,20]. Als3, a molecular mimic of mammalian cadherins, facilitates *C. albicans* adhesion and invasion of human umbilical vein endothelial and oral epithelial cells [21]. Based on the important roles of the cell wall in *C. albicans* colonization and invasion, we propose that understanding the key factors and regulatory processes of cell wall remodeling will be helpful for discovering new antifungal targets and developing novel antifungal drugs.

Cas5, a transcriptional regulator of cell wall integrity, is activated to promote *C. albicans* cell wall remodeling in the event of cell wall damage through dynamic changes in cell wall components [16,22]. For example, Cas5 responds to cell wall stress from echinocandins and regulates more than 60% of the gene’s response to echinocandins through interactions with the virulence-associated transcription factor Efg1 [23,24]. Moreover, the depletion of Cas5 reduces the resistance of strains carrying mutations in *FKS1*, a well-accepted echinocandin target gene [23]. Cas5 also contributes to *C. albicans* survival under cell wall disturbances induced by congo red, sodium dodecyl sulfate, calcofluor white (CFW), and low temperature [23,25,26]. More importantly, Cas5 lacks an identifiable ortholog in most other eukaryotes [9]. Collectively, these findings suggest that Cas5 regulates cell wall remodeling in multiple ways to promote *C. albicans* survival, highlighting the potential of Cas5 for *C. albicans* pathogenicity. In addition to its role in the response to cell wall stress, Cas5 is also involved in the regulation of *C. albicans* virulence. The depletion of Cas5 contributed to the improved survival of *Toll* mutant flies, *Caenorhabditis elegans*, and BALB/c mice when they suffered from disseminated candidiasis [27,28]. Additionally, it has been reported that the Cas5 null mutant exhibited filamentation deficiency in *C. elegans* and decreased fungal burden in the kidneys and spleen of BALB/c mice [27,28]. In addition to these findings in vivo, a significantly reduced adhesion activity of the Cas5 null mutant on a silica gel surface was also observed in vitro [29].

Recent studies have highlighted the importance of Cas5 in responding to cell wall stress and regulating *C. albicans* virulence. However, the specific links of Cas5 in the cell wall remodeling process or the relationships between Cas5-modulated cell wall remodeling and vital *C. albicans* virulence factors remain underexplored. This study aims to investigate the role of Cas5 in regulating cell wall remodeling and the effects on *C. albicans* virulence and host interactions. By examining Cas5-mediated β-glucan masking, cell surface hydrophobicity (CSH) alteration, and the differential expression of CWPs, Cas5 was hypothesized to participate in regulating innate immune cells recruitment and the adhesion process to host cells, thereby influencing the colonization and invasion of *C. albicans* in OPC pathogenesis.

## 2. Materials and Methods

### 2.1. Study Design

#### 2.1.1. Type of Study

This study is a laboratory-based experimental study designed to investigate the effects of Cas5 depletion in *C. albicans* on cell wall structure, adhesion to host cells, morphology, and innate immune cells recruitment in a BALB/C mouse model of oropharyngeal candidiasis (OPC).

#### 2.1.2. Key Variables

(1) Independent Variables
  (a) Cas5 Depletion: presence or absence of the *CAS5* gene (wild-type *C. albicans SC5314* [WT] vs. Cas5 null mutant [*cas5Δ/Δ*]).
(2) Dependent Variables
  (a) Cell Wall Structure: changes in *C. albicans* cell wall glycans exposures and CWPs expressions;
  (b) Adhesion to Host Cells: assessment of the ability of *C. albicans* to adhere to host endothelial and epithelial cells;
  (c) Morphology: assessment of the ability of hypha and biofilm formation;
  (d) Innate Immune Cells Recruitment: quantification of neutrophils and macrophages infiltration in the tongue tissues of BALB/C male mice with OPC.

#### 2.1.3. Sources of Variation

To ensure the reliability and reproducibility of our findings, we carefully documented and controlled for potential sources of variation:

(1) Strain Variability
  (a) Genetic Background: both WT and *cas5Δ/Δ* strains were derived from the same parental strain (*SC5314*) to minimize genetic background differences;
  (b) Knockout Efficiency: the efficiency of the *CAS5* gene knockout was confirmed using PCR and sequencing to ensure consistent genetic modification across all experimental samples;
  (c) Complementation Efficiency: the efficiency of the *CAS5* gene complementation was confirmed using RT-qPCR to ensure consistent genetic modification across all experimental samples.
(2) Culture Conditions
  (a) Medium: all cultures were grown in YPD medium at 30 °C or RPMI 1640 medium containing 10% FBS with constant shaking (220 rpm) to maintain consistent growth conditions;
  (b) Growth Phase: experiments were conducted using *C. albicans* cells harvested at late-log phase to ensure uniformity in metabolic activity and physiological state.
(3) Mouse Model Variability
  (a) All the BALB/C mice used in this study were male, aged 6–8 weeks, and maintained in identical baseline health status.
(4) Random Error
  (a) Replicate Measurements: each experiment was repeated at least three times, and the average value was taken as the final result to reduce the impact of random errors.

#### 2.1.4. Study Design Overview

The study design will follow a structured approach to address the research objectives:

(1) Strain Construction
  (a) Generation of *C. albicans* strains with and without Cas5 depletion using CRISPR/Cas9 homologous recombination technology.
(2) In Vitro Experiments
  (a) Cell Wall Analysis: use transmission electron microscopy, flow cytometry, fluorescence microscopy, TMT-labeled proteomics, and CSH assay to evaluate changes in cell wall structure induced by Cas5 depletion;
  (b) Adhesion Assays: conduct adhesion assays using typical endothelial and epithelial host cells to measure the adhesion capacity of various *C. albicans* strains;
  (c) Morphological Transition Assessment: employ fluorescence microscopy, scanning electron microscopy, and biofilm formation assay to examine morphology transition of various *C. albicans* strains.
(3) In Vitro Experiments
  (a) Mouse Model Establishment: infect BALB/C mice with various *C. albicans* strains via oral inoculation to establish OPC;
  (b) Innate Immune Cell Recruitment: collect tongue tissues at first day post-infection and quantify immune cell infiltration using immunofluorescence staining;
  (c) *C. albicans* pathogenicity: collect tongue tissues at the fifth day post-infection and evaluate the pathogenicity of various *C. albicans* strains using histopathologic examination, immunohistochemistry (IHC), and fungal burden.
(4) Statistical Analysis

All data were plotted and analyzed for statistical significance using GraphPad Prism v.9.3.1. Data were compared using one-way analysis of variance (ANOVA) or Student’s *t* test. The graphs are annotated to indicate the levels of statistical significance of the results (* *p* < 0.05, ** *p* < 0.01, *** *p* < 0.001, and **** *p* < 0.0001).

### 2.2. Plasmid and Strain Constructions

The oligonucleotides and primers used for constructing and detecting plasmids are listed in Table S1. Wild-type *C. albicans SC5314* (WT) was used to generate Cas5 null mutant (*cas5Δ/Δ*). The pV1093 plasmid (Addgene plasmid #111428), a gift from Gerald Fink, was used to construct a *C. albicans* CRISPR system targeting the *CAS5* gene to create *cas5Δ/Δ* (Figure S1) [30]; the details are presented in the Supplementary Materials. Disruption of *CAS5* was confirmed by PCR and sequencing. To generate a complemented strain expressing *CAS5* under the MET3 promoter (*cas5Δ/Δ + CAS5*), *CAS5* ORF (*CAS5* guide sequence was replaced by their synonymous codons) was cloned into a modified pCaEXP plasmid containing *RP10* locus, *MET3* promoter, and zeocin resistance sequence [31]. The construct was transformed into *cas5Δ/Δ* to generate the *CAS5* complemented strain.

### 2.3. Medium and Growth Conditions

*C. albicans* strains were stored in 25% glycerol stocks at −80 °C. Subsequently, routine cultivation was accomplished in YPD medium (Solarbio, Beijing, China) at 30 °C (yeast-form). For hypha induction, *C. albicans* strains were grown in RPMI 1640 medium containing 10% fetal bovine serum (FBS) (Gibco, New York, NY, USA) at 37 °C (hypha-form). Host cells, human umbilical vein cells EA. hy926 and mouse fibroblasts L-929 cells (ATCC CRL-2922 and ATCC CCL-1), were cultured in DMEM medium (United Bioresearch, Maroota, NSW, Australian) containing 10% FBS at 37 °C with 5% CO_2_.

### 2.4. Transmission Electron Microscopy (TEM) of Cell Wall Structure

Yeast-form and hypha-form *C. albicans* grown for 90 min were collected and fixed with 2.5% glutaraldehyde and 2% osmium tetroxide for 2 h. After dehydration through a graded series of ethanol, the samples were embedded in an Epon resin. The samples were sliced into ultrathin sections and observed by TEM (Hitachi HT7800, Tokyo, Japan) [32]. Then, the thickness of the cell wall β-glucan in three representative areas per cell for each strain was counted using ImageJ 1.51j8.

### 2.5. Cell Wall Glycans Exposures

Yeast-form and hypha-form *C. albicans* grown for 90 min were incubated with aniline blue (1 mg/mL, Macklin, Shanghai, China) for 5 min at room temperature, which specifically recognizes β-glucan [33]. The cell wall mannan and chitin of yeast-form cells grown in YPD medium at 30 °C were stained with 200 μg/mL of FITC-conjugated concanavalin A (ConA-FITC) and FITC-conjugated wheat germ agglutinin (WGA-FITC) (Sigma-Aldrich, St. Louis, MO, USA), respectively. Additionally, cell wall mannan exposures were also viewed under a fluorescence microscope (ZEISS Axio Vert A1, Oberkochen, Germany). All samples were examined using flow cytometry (BD FACSCelesta, Fremont, CA, USA, for the detection of β-glucan exposure; CytoFLEX Beckman Coulter, Brea, CA, USA, for the detection of mannan and chitin exposures). Flow cytometry parameters were as follows: (1) For β-glucan exposure detection, voltages were set as FSC-A 709 V, SSC-A 390 V, and Brilliant violet 786 (BV786) 590 V. The BV786 fluorophore was excited by the violet laser (405 nm), and its emitted fluorescence was detected through the BV786 channel at a flow rate of 50 μL/min; (2) For mannan exposure detection, voltages were adjusted to FSC-A 45 V, SSC-A 45 V, and FITC 1 V. The FITC fluorophore was excited by the blue laser (488 nm), and its emitted fluorescence was detected through the FITC channel at a flow rate of 60 μL/min; (3) For chitin exposure detection, voltages were set as FSC-A 35 V, SSC-A 25 V, and FITC 1 V. The FITC fluorophore was excited by the blue laser (488 nm), and its emitted fluorescence was detected through the FITC channel at a flow rate of 60 μL/min. Data for 10,000 events were collected. All assays were performed in triplicates.

### 2.6. Expression of Cell Wall Proteins (CWPs)

The expression levels of *C. albicans* CWPs were determined by TMT-labeled proteomics. SDT (4% SDS, 100 mM Tris-HCl, 1 mM DTT, pH 7.6) buffer was used for yeast-form *C. albicans* cells (WT and *cas5Δ/Δ* grown in YPD medium at 30 °C) lysis and protein extraction. After being quantified with BCA Protein Assay Kit (Bio-rad, Hercules, CA, USA), proteins were digested using trypsin. The digested peptides of each sample were desalted on C18 Cartridges (Empore SPE Cartridges C18, bed I.D. 7 mm, volume 3 mL, Sigma) and reconstituted in 0.1% (*v*/*v*) formic acid. One hundred micrograms of peptide mixture from each sample was labeled using TMT reagent (Thermo Scientific, Waltham, MA, USA) and then fractionated by SCX chromatography using the AKTA Purifier system (GE Healthcare, Buckinghamshire, UK). The fractionated peptides were analyzed using LC-MS/MS on a Q Exactive mass spectrometer (Thermo Scientific) coupled with an Easy nLC system (Thermo Scientific). Peptides were loaded onto a reverse-phase trap column (Thermo Scientific Acclaim PepMap 100, 100 μm × 2 cm, nanoViper C18) connected to a C18-reversed phase analytical column (Thermo Scientific Easy Column, 10 cm long, 75 μm inner diameter, 3 μm resin) using buffer A (0.1% formic acid). Separation was performed with a linear gradient of buffer B (84% acetonitrile and 0.1% formic acid) at a flow rate of 300 nL/min controlled by IntelliFlow technology. The mass spectrometer was operated in positive ion mode. MS data were acquired using a data-dependent top10 method dynamically choosing the most abundant precursor ions from the survey scan (300–1800 *m*/*z*) for HCD fragmentation. Automatic gain control (AGC) target was set to 3e6, and maximum inject time to 10 ms. Dynamic exclusion duration was 40.0 s. Survey scans were acquired at a resolution of 70,000 at *m*/*z* 200 and resolution for HCD spectra was set to 17,500 at *m*/*z* 200, and isolation width was 2 *m*/*z*. Normalized collision energy was 30 eV and the underfill ratio, which specifies the minimum percentage of the target value likely to be reached at maximum fill time, was defined as 0.1%. The instrument was run with peptide recognition mode enabled. Differentially expressed proteins were locally searched using NCBI BLAST+client software (ncbi-blast-2.2.28+-win32.exe), and homologous sequences were found using InterProScan 5.59-91.0. Gene ontology (GO) terms were mapped and annotated using Blast2GO 6.0.1. GO annotation results were plotted using R scripts. These proteins were also BLASTed against the online Kyoto Encyclopedia of Genes and Genomes (KEGG) database (http://geneontology.org/, accessed on 20 August 2023) to retrieve their KEGG orthologs and subsequently mapped to KEGG pathway [34]. All assays were performed in triplicates.

### 2.7. CSH Assay

*C. albicans* grown overnight in YPD medium at 37 °C or room temperature (25 °C) was collected and then resuspended in PBS to an optical density of 0.4 at 492 nm. 1 mL of xylene was then added to 3 mL of each suspension. After vortex mixing for 1 min, the mixtures were left for 5 min until the two phases were separated [35]. The relative CSH was expressed as the percentage reduction in the initial turbidity of the aqueous suspension. All assays were performed in triplicates.

### 2.8. Adhesion on Host Cells

Human umbilical vein cells EA. hy926 and mouse fibroblasts L-929 cells, which are typical endothelial and epithelial cells, were used to establish a *C. albicans*–host cell co-incubation model [36]. Briefly, EA. hy926 and L-929 cells labeled with 1,1′-dioctadecyl-3,3,3′,3′-tetramethylindocarbocyanine perchlorate (DiI, 5 μM, Beyotime, Shanghai, China) grew to 80–90% confluency in 48-well plates and then inoculated with yeast-form *C. albicans* (2.5 × 10^6^ cells) stained with CFW (10 μM, Sigma-Aldrich). The infected cells were incubated in FBS-free RPMI 1640 medium under 5% CO_2_ at 37 °C. After a certain incubation time (0.5, 1.5, and 2 h), the medium was discarded, and non-adherent *C. albicans* cells were removed. Cells were fixed with 4% paraformaldehyde and viewed under a fluorescence microscope (ZEISS Axio Vert A1, Oberkochen, Germany). All assays were performed in triplicates.

### 2.9. Fluorescence Microscopy of C. albicans Hypha Growth

Yeast-form *C. albicans* was harvested and adjusted to 1 × 10^5^ cells/mL of final concentration with RPMI 1640 medium containing 10% FBS (hypha-induction condition). The cultures were incubated at 37 °C for 24 h. At 0, 2, 5, 7, and 24 h, aliquots were added to glass slides and stained with CFW (10 μM, Sigma-Aldrich). Images were captured using a fluorescence microscope (ZEISS Axio Vert A1), and hypha frequencies in three representative areas were counted using the ImageJ 1.51j8.

### 2.10. Scanning Electron Microscopy (SEM) of C. albicans Hypha Growth

Yeast-form *C. albicans* was prepared as a suspension in RPMI 1640 medium containing 10% FBS (hypha-induction condition) at a concentration of 1 × 10^7^ cells/mL. The suspension was added to 24-well plates containing cell slides and incubated at 37 °C for 90 min. The cells were fixed with 2.5% glutaraldehyde followed by 1% osmic acid. The dehydration was performed using a graded series of ethanol solutions. After coating with gold, cell morphology was observed using SEM (Hitachi SU8100, Tokyo, Japan).

### 2.11. Biofilm Formation Assay

Biofilm formation assay was performed according to the method proposed by Baohua Xu and colleagues with some modification [37]. Yeast-form *C. albicans* was prepared as a suspension in RPMI 1640 medium containing 10% FBS (biofilm-induction condition) at a concentration of 2 × 10^6^ cells/mL in 96-well plates and incubated at 37 °C for 48 h. Cells attached to the biofilm surface were removed by washing with PBS. After being fixed with methanol for 15 min, 100 μL of 0.2% crystal violet (CV) (Beyotime) was added to the wells and incubated for 20 min. Excess CV was washed with PBS, and the plates were air-dried for 30 min; 100 μL of ethanol was used to dissolve the CV in every well. The biomass was quantified by measuring the absorbance of the CV solution at 600 nm using a microplate reader (Thermo Multiskan MK3, Waltham, MA, USA). All assays were performed in triplicates.

### 2.12. Mouse Model of C. albicans Infection

For the mouse model of OPC [38], BALB/c male mice (n = 13 per group, aged 6–8 weeks) were randomly assigned to four groups. The feeding methods for the mice in each group were consistent with those used in the above model. Mice were immunosuppressed by subcutaneous administration of cortisone acetate (225 mg/kg) (Aladdin, Shanghai, China) every other day until the end of the experiment. Twenty-four hours after the first immunosuppression, mice were anesthetized by intraperitoneal injection of tribromoethanol (0.6 mg/10 g) (TCI, Shanghai, China). Swabs soaked in *C. albicans* suspension (yeast-form WT/*cas5Δ/Δ*/*cas5Δ/Δ + CAS5*) at a concentration of 2 × 10^7^ cells/mL for 5 min were placed under the mouse’s tongue for 75 min, and HBSS was used as a control. On the first day (neutrophil and macrophage recruitment assay) and fifth day post-infection (Histopathologic examination, IHC, and fungal burden), respectively, the mice were euthanized, and tongue tissues were used for subsequent examinations.

### 2.13. Neutrophil and Macrophage Recruitment Assay

The ability of *C. albicans* strains to recruit neutrophils and macrophages was evaluated in the mouse models of OPC described above. Immunofluorescence staining was used to evaluate phagocyte recruitment in tongue tissues. Briefly, tongue tissues were randomly selected from each group and fixed with 4% paraformaldehyde. Tongue tissues (n = 3 per group) were embedded in wax, and 5 μm thick tissue sections were prepared using a microtome. Tissue sections were deparaffinized in xylene and rehydrated through a graded ethanol series. Antigen retrieval was performed by boiling the sections in citrate buffer (pH 6.0) for 10 min. After blocking with 5% bovine serum albumin (BSA) for 1 h at room temperature, the sections were incubated overnight at 4 °C with primary antibodies (anti-Ly6G rabbit (1:200) and anti-F4/80 rabbit (1:500)) to label neutrophils and macrophages, respectively (Servicebio, Wuhan, China). Subsequently, the sections were incubated with an Alexa Fluor 488/549 goat anti-mouse IgG antibody. After counterstaining with 4,6-diamidino-2-phenylindole (DAPI) (Servicebio), the slides were imaged using a fluorescence microscope (Nikon Eclipse Cl, Tokyo, Japan), and then the average fluorescence intensity of these samples (whole area of tongue tissues) from three mice of each group were quantified by ImageJ 1.51j8.

### 2.14. Histopathologic Examination, IHC, and Fungal Burden

Tongue tissue sections from a mouse model of OPC were used for pathological examination by H&E and PAS staining. IHC of these sections were performed by incubating with corresponding primary antibodies (rabbit anti-TNF-α (1:400), rabbit anti-IL-1β (1:800), rabbit anti-IL-6 (1:300), or rabbit anti-IL-10 (1:800) (Servicebio) overnight at 4 °C. The membranes were then incubated with HRP and goat anti-rabbit IgG (Servicebio). The average optical density (AOD) of randomly selected areas of tongue tissues from each group (n = 3 per group) were quantified by ImageJ 1.51j8. Immunostaining was enhanced with 3,3′-diaminobenzidine and counterstained with hematoxylin. Stained images were captured using a microscope (ZEISS Axio Vert A1). To determine the fungal burden, the tongue tissues of the remaining mice from each group (n = 13 per group) were harvested, weighed, separately homogenized with a tissue grinder, and quantitatively cultured by incubating serial dilutions on YPD agar plates (containing 50 μg/mL carbenicillin).

## 3. Results

### 3.1. Cas5 Helps Cell Wall β-Glucans Mask

**Figure 1:**
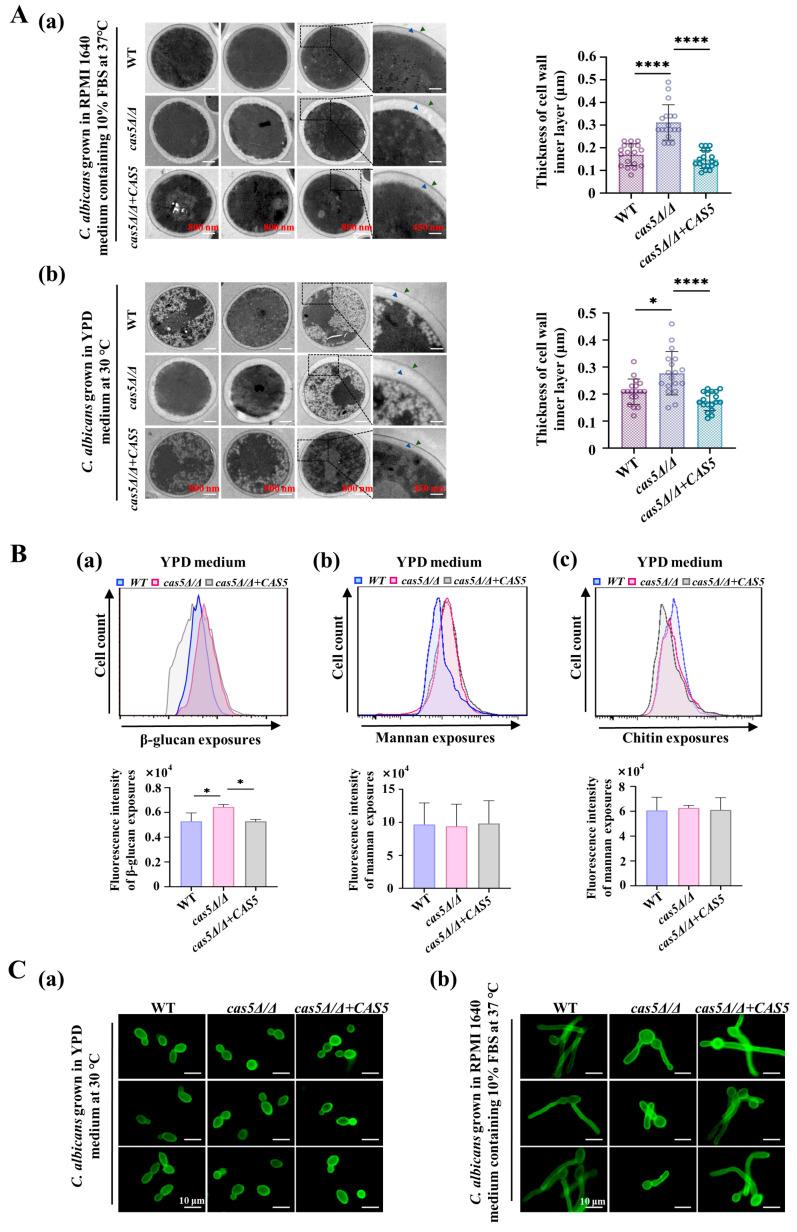
Cas5 mediates biosynthesis and distribution of cell wall glycans of *C. albicans*. (**A**). TEM images (**left**) and quantitative analysis of cell wall inner layer thickness (**right**) in various *C. albicans* strains. (**a**): *C. albicans* grown in YPD medium at 30 °C overnight and subsequently cultured in RPMI 1640 medium containing 10% FBS at 37 °C for 90 min; (**b**): *C. albicans* grown in YPD medium at 30 °C overnight and subsequently cultured in YPD medium at 30 °C for 90 min. Mannan layer: area indicated by green arrows; inner layer (including β-glucan and chitin): area indicated by blue arrows. Measurements were analyzed using one-way ANOVA (WT VS. *cas5Δ/Δ*, WT VS. *cas5Δ/Δ* + *CAS5*, and *cas5Δ/Δ* VS. *cas5Δ/Δ* + *CAS5*); Asterisks show statistically significant differences (*, *p* < 0.05; ****, *p* < 0.0001). (**B**). Flow cytometry analysis of cell wall glycans exposures in various *C. albicans* strains grown in YPD medium at 30 °C overnight and subsequently cultured in YPD medium at 30 °C for 90 min (**above**). (**a**): Cell wall β-glucan; (**b**): cell wall mannan; and (**c**): cell wall chitin. Histogram plots (**below**) are representative of data collected in three independent replicate experiments, with error bars; measurements were analyzed using one-way ANOVA (WT VS. *cas5Δ/Δ*, WT VS. *cas5Δ/Δ* + *CAS5*, and *cas5Δ/Δ* VS. *cas5Δ/Δ* + *CAS5*); asterisks show statistically significant differences (*, *p* < 0.05). (**C**). Fluorescence micrographs of cell wall mannan exposures in various *C. albicans* strains. (**a**): *C. albicans* grown in YPD medium at 30 °C overnight and subsequently cultured in YPD medium at 30 °C for 90 min.; (**b**) *C. albicans* grown in YPD medium at 30 °C overnight and subsequently cultured in RPMI 1640 medium containing 10% FBS at 37 °C for 90 min; ConA-FITC: staining cell wall mannan. Bar: 10 μm.

To systematically elucidate the role of Cas5 in cell wall remodeling, we aimed to determine the regulatory effects of Cas5 on essential components of the *C. albicans* cell wall, including polysaccharides and virulence-related CWPs. Here, we created a *cas5Δ/Δ* null mutant using CRISPR/Cas9 in the WT strain (*SC5314*). To generate reintegrant control strains, we complemented *cas5Δ/Δ* with *CAS5* ORF under the control of the strong *C. albicans* MET3 promoter (Figures S1 and S2). Subsequently, the effect of Cas5 on the *C. albicans* cell wall structure was evaluated using TEM. Obviously, *cas5Δ/Δ* exhibited a significantly thicker inner layer (including β-glucan and chitin, indicated by blue arrows), and the average thickness was 1.3-fold and 1.8-fold greater than that in the control strains incubated in the YPD medium and the RPMI 1640 medium containing 10% FBS, respectively (Figure 1A and Figure S3A,B). Additionally, we did not observe obvious differences in the mannan layers of the three strains under the two culture conditions. Based on these results, we propose that the thickened inner glycans in *cas5Δ/Δ* may also lead to the increased exposure of them in the cell wall. Thus, we examined the exposure of *C. albicans* to cell wall glycans using flow cytometry (Figure 1B). The *cas5Δ/Δ* cultured in the YPD medium (Figure 1B(a)) or in the RPMI 1640 medium (Figure S3C) showed a significant increase in β-glucan exposure than that in the WT and *cas5Δ/Δ + CAS5* strains. There were no differences in exposure to chitin or mannan between the tested strains cultured in the YPD medium (Figure 1B). In addition, the fluorescence images also demonstrated a consistent level of cell wall mannan exposures among the tested strains cultured in the YPD medium or in RPMI 1640 medium (Figure 1C). Overall, we propose that Cas5 promotes cell wall β-glucan masking.

### 3.2. Cas5 Regulates CWP Expression

**Figure 2:**
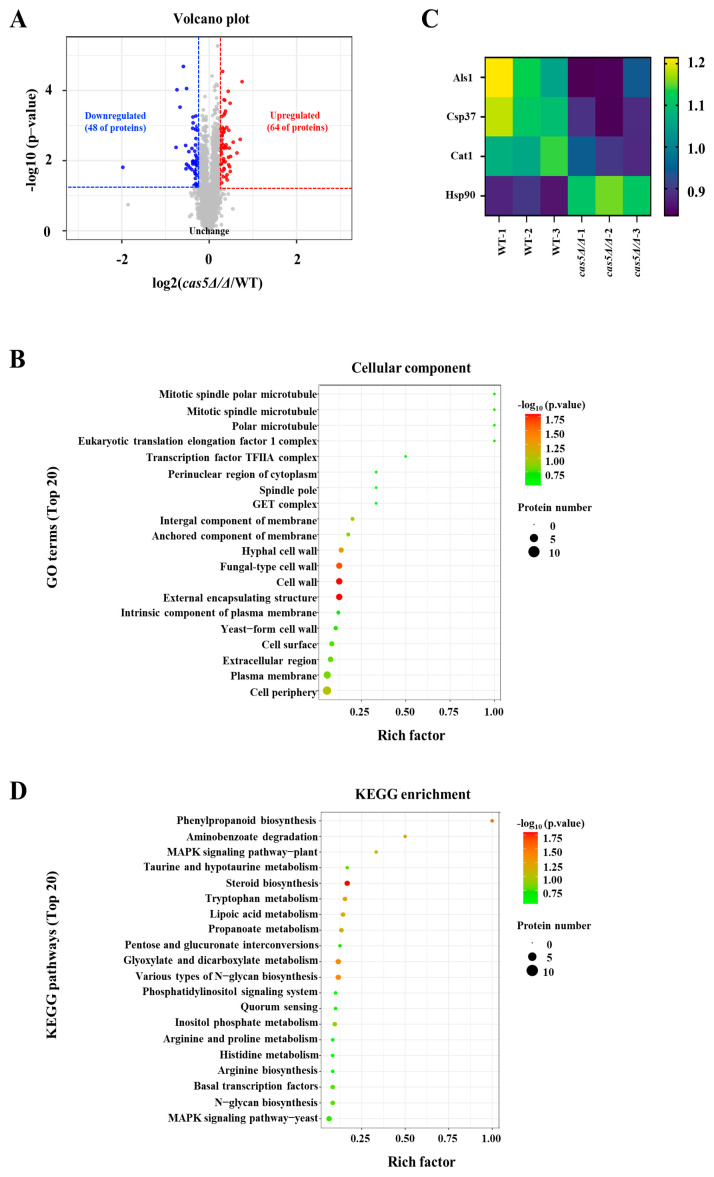
Cas5 mediates differential expressions of *C. albicans* cell wall proteins. (**A**). Volcano plot of differential-expression proteins in *cas5Δ/Δ* mutant relative to WT strain analyzed by TMT-labeled proteomics (*p* < 0.05). (**B**). Bubble diagram of GO enrichment of top 20 significantly differentially expressed proteins between *cas5Δ/Δ* and WT. All terms have *p* value of <0.05. (**C**). Heat map depicting virulence-related protein expression levels in *cas5Δ/Δ* compared to WT. (**D**). Bubble diagram of KEGG pathway analysis of top 20 significantly differentially expressed proteins in *cas5Δ/Δ* compared to WT. All terms have *p* value < 0.05. All data were determined in three biological replicates.

Apart from the increased β-glucan exposure, the TEM images also showed obviously less electron density in the cell wall of *cas5Δ/Δ*, which may be due to the change in CWPs. Here, TMT-labeled proteomics analysis was conducted to examine the proteins whose expression differed significantly between the *cas5Δ/Δ* and WT strains. A volcano plot showed that the deletion of Cas5 triggered notable changes in the overall protein expression of *C. albicans*, and 112 significantly upregulated and downregulated proteins were identified (*p* < 0.05) (Figure 2A). The GO term analysis revealed that, upon the deletion of Cas5 the sets of differentially expressed proteins were significantly enriched in the hypha-form and yeast-form cell wall components (*p* < 0.05) (Figure 2B) and were involved in multiple virulence-related CWPs (Figure 2C). Among them, both adhesion-related proteins, Als1 and Csp37, and the hypha formation-related protein, Cat1, were significantly downregulated in *cas5Δ/Δ* [39,40,41,42]. Moreover, we also found in *cas5Δ/Δ* that Hsp90, which has hyphal inhibitory activity in the active state, was upregulated [43]. These results suggest that the depletion of Cas5 may lead to a deficiency in adhesion and hyphal development. Further KEGG pathway analysis revealed that Cas5 is mainly involved in the biosynthesis of various types of N-glycans (Figure 2D). In conclusion, Cas5 also mediates cell wall remodeling by regulating the expression of the CWPs associated with adhesion and hypha development.

### 3.3. Cas5 Regulates C. albicans Adhesion to Host Cells, Morphology Transition, and Cell Surface Hydrophobicity (CSH)

**Figure 3:**
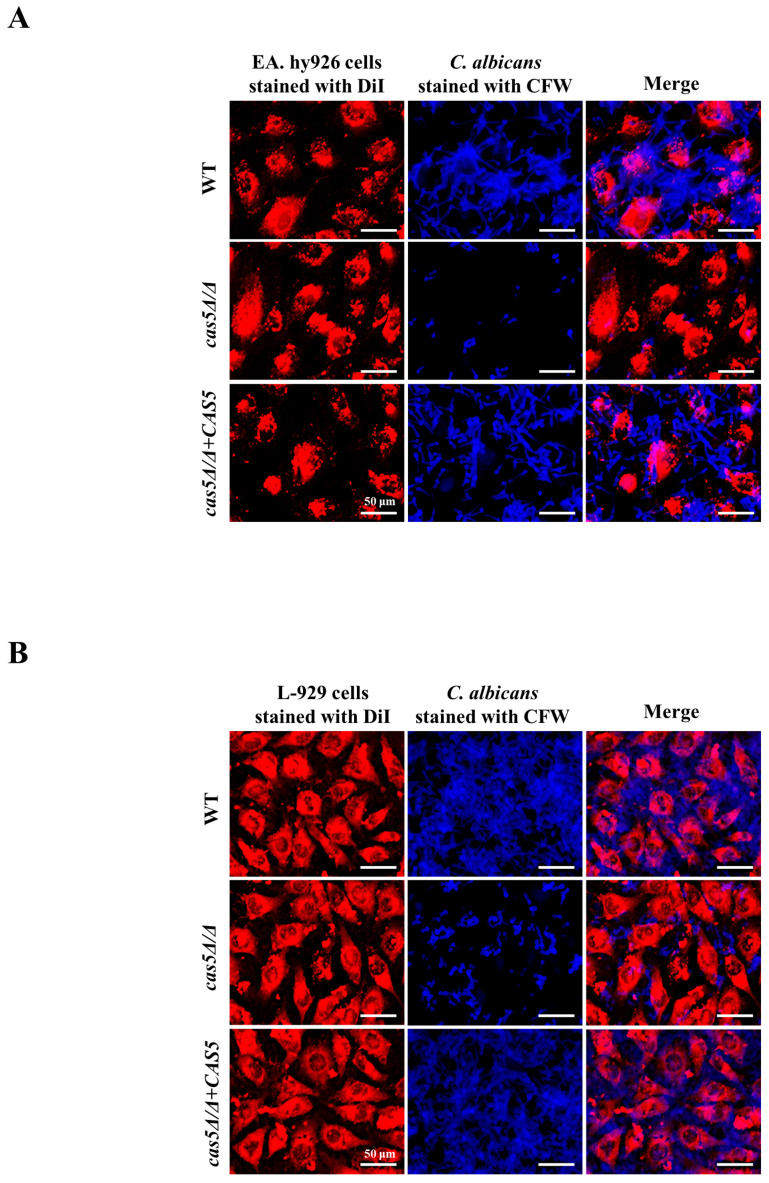
Cas5 promotes *C. albicans* adhesion to host cells. (**A**). Fluorescence micrographs of adhesion ability of yeast-phase *C. albicans* to EA. hy926 cells after 120 min incubation in RPMI 1640 medium at 37 °C. (**B**). Fluorescence micrographs of adhesion ability of yeast-phase *C. albicans* strains to L-929 cells after 120 min incubation in RPMI 1640 medium at 37 °C. Calcofluor white (CFW): staining *C. albicans*, DiI: staining EA. hy926 cells. Bar: 50 μm.

**Figure 4:**
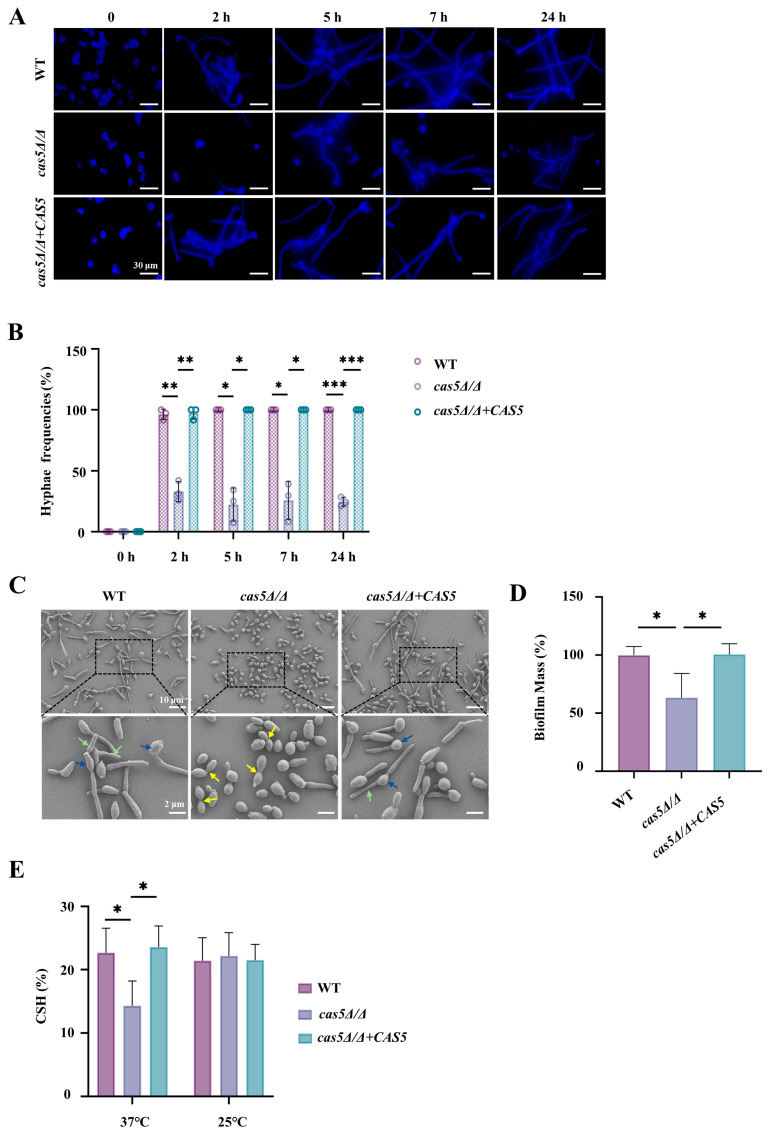
Cas5 contributes to *C. albicans*’s morphology transition from yeast to hypha and regulates cell surface hydrophobicity of *C. albicans*. (**A**). Fluorescence micrographs of *C. albicans* strains at different time points cultured at 37 °C in RPMI 1640 medium containing 10% FBS; *C. albicans* stained by calcofluor white (CFW). (**B**). Histogram plots are representative of statistical results of hypha frequencies in *C. albicans* strains, with error bars. Measurements were analyzed using one-way ANOVA (WT VS. *cas5Δ/Δ*, WT VS. *cas5Δ/Δ* + *CAS5*, and *cas5Δ/Δ* VS. *cas5Δ/Δ* + *CAS5*); asterisks show statistically significant differences (*, *p* < 0.05; **, *p* < 0.01; ***, *p* < 0.001). (**C**). SEM images of morphologies of various *C. albicans* strains after 90 min cultured in RPMI 1640 medium containing 10% FBS at 37 °C. Obvious constrictions at septal sites of *cas5Δ/Δ* cells are indicated by yellow arrows. Apical and subapical cells of WT and *cas5Δ/Δ + CAS5* are indicated by blue and green arrows, respectively. (**D**). Biofilm formation assay of *C. albicans* strains incubated in RPMI 1640 medium containing 10% FBS at 37 °C for 48 h, biofilm mass was quantified by a crystal violet (CV) assay. All data were in three independent replicate experiments and analyzed using one-way ANOVA (WT VS. *cas5Δ/Δ*, WT VS. *cas5Δ/Δ* + *CAS5*, and *cas5Δ/Δ* VS. *cas5Δ/Δ* + *CAS5*); asterisks show statistically significant differences (*, *p* < 0.05). (**E**). CSH of yeast-phase *C. albicans* strains grown in YPD medium at 37 °C and room temperature. All data were in three independent replicate experiments and analyzed using one-way ANOVA (WT VS. *cas5Δ/Δ*, WT VS. *cas5Δ/Δ* + *CAS5*, and *cas5Δ/Δ* VS. *cas5Δ/Δ* + *CAS5*); asterisks show statistically significant differences (*, *p* < 0.05).

The effects of Cas5 on cell wall β-glucan and CWPs implied that Cas5 may promote *C. albicans* adhesion to host cells. Here, we examined the adhesion ability of *C. albicans* to host cells in a model in which *C. albicans* was incubated with either the endothelial cells EA.hy926 or epithelial cells L-929 for 120 min. Representative fluorescence images at different time points showed that, compared with those of the control strains, the number of *cas5Δ/Δ* that adhered to both the host cells, EA. hy926 and L-929, decreased (Figure 3, Figures S4 and S5); moreover, most *cas5Δ/Δ* cells were still in the yeast form, whereas the WT and *cas5Δ/Δ + CAS5* strains underwent a morphological transition from yeasts to hypha. Therefore, we studied the regulatory effect of Cas5 on hypha formation and development in detail. The yeast-to-hypha differentiation of the three tested *C. albicans* strains, induced by the RPMI 1640 medium supplemented with 10% FBS at 37 °C, was monitored. CFW-stained images showed that the hypha of the WT and *cas5Δ/Δ + CAS5* strains formed within 2 h of induction and their lengths continued to increase with extended induction time (Figure 4A). However, the depletion of Cas5 significantly impacted hyphal growth in 2 h (Figure 4A). After 2 h, although *cas5Δ/Δ* did not show a complete defect of hypha formation, its hypha frequencies were significantly lower than the WT and *cas5Δ/Δ + CAS5* strains (Figure 4A,B). The SEM images taken following 90 min of hypha induction showed that the majority of the *cas5Δ/Δ* strains exhibited yeast forms, and certain individual cells exhibited pseudohyphal characteristics, obvious constrictions at the septal sites of cells (yellow arrows), and nearly synchronous cell divisions (Figure 4C). In comparison to this phenotype, both the WT and *cas5Δ/Δ + CAS5* strains exhibited clear hypha features, characterized by indistinct septation points and notably delayed subapical cell division relative to their apical counterparts (Figure 4C). Based on these findings, it becomes evident that Cas5 plays an essential role in facilitating the growth and development of *C. albicans* hypha.

Moreover, hypha development promoted *C. albicans* biofilm formation; thus, we analyzed the biofilm mass of the three tested strains, and our analysis revealed a significant reduction in biofilm mass among the *cas5Δ/Δ* mutants compared to the WT and *cas5Δ/Δ + CAS5* controls (Figure 4D and Figure S6). As hypha can further strengthen *C. albicans* adhesion and invasion [44], we propose that the observed decrease in adhesive strength in *cas5Δ/Δ* is closely related to Cas5-mediated morphological switching.

Together, CSH and CWPs influence the adhesion ability of *C. albicans* [45]. Our proteomic analysis revealed that Cas5 positively correlated with *C. albicans* adhesion proteins (Figure 2). Here, we focused on the effects of Cas5 on CSH. Considering that CSH status is related to the cell wall polysaccharides [46] and the findings on the regulatory role of Cas5 in cell wall polysaccharides (Figure 1), we measured the effect of Cas5 on the CSH of yeast *C. albicans* in the YPD medium at 37 °C or at room temperature (Figure 4E). Compared to the control strains, the CSH of the strains in which *cas5Δ/Δ* was incubated in the YPD medium at 37 °C were significantly lower, while the CSH of the three tested strains cultured at room temperature showed no significant difference (Figure 4E). These results suggest that CSH regulation by Cas5 is influenced by temperature, indicating that Cas5-mediated CSH is an important factor for *C. albicans* adhesion in vivo.

### 3.4. Cas5 Knockout Results in Increased Neutrophil Recruitment

**Figure 5:**
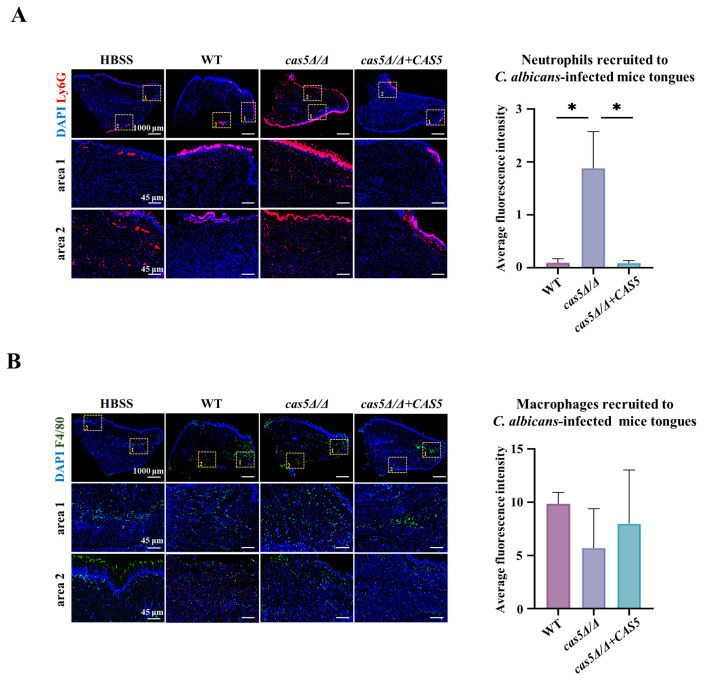
Cas5 modulates host innate immunity. (**A**). Fluorescence micrographs (**left**) and quantitative analysis (**right**) of neutrophils recruitment in *C. albicans* strain-infected mice tongue tissues. (**B**). Fluorescence micrographs (**left**) and quantitative analysis (**right**) of macrophages recruitment in *C. albicans* strain-infected mice tongue tissues. DAPI: staining DNA to localize cells, rabbit anti-ly6G antibody: neutrophils labeling, rabbit anti-F4/80 antibody: macrophages labeling. One sample is shown for each group. Representative magnification areas with enriched recruitment of neutrophils and macrophages, respectively, are shown for each group. The sterile HBSS is used as control. Bar: 1000 μm (DAPI F4/80 Ly6G), 45 μm (area 1 and area 2). Measurements were analyzed using one-way ANOVA (WT VS. *cas5Δ/Δ*, WT VS. *cas5Δ/Δ* + *CAS5*, and *cas5Δ/Δ* VS. *cas5Δ/Δ* + *CAS5*); asterisks show statistically significant differences (*, *p* < 0.05).

Based on the ability of host phagocytes to recognize *C. albicans* cell wall β-glucan [13], we hypothesized that Cas5 helps *C. albicans* to prevent the recognition of host innate immunity by masking β-glucan in the cell wall. To verify our hypothesis, the ability of the WT, *cas5Δ/Δ*, and *cas5Δ/Δ + CAS5* strains to recruit innate immune cells was evaluated in the mouse model of OPC. On the first day, post-sublingual infection in the mouse model of OPC, the mice were sacrificed, and their tongues were excised for innate immune cell recruitment analysis using immunofluorescence staining. As we thought, compared with the WT-infected mice or *cas5Δ/Δ + CAS5*-infected mice, those infected with *cas5Δ/Δ* showed more neutrophils in their tongues (Figure 5A and Figure S7B), and there was no significant difference in the recruitment of macrophages across the tested strains (Figure 5B and Figure S7B). These results suggest that Cas5 helps *C. albicans* block the recognition of neutrophils. Generally, hypha-form *C. albicans* displays more β-glucan exposure compared to its yeast form; this increased exposure should theoretically stimulate the recruitment of additional phagocytes [47]. However, this study uncovered an intriguing observation—the *cas5Δ/Δ* with defective hypha development had a stronger ability to recruit neutrophils than the WT and *cas5Δ/Δ + CAS5* with normal hyphal development (Figure 5A). Thus, there is a good correlation between the Cas5-mediated cell wall β-glucan exposure and the effectiveness at attracting neutrophils towards specific regions within animals’ mouths. These findings provide valuable insights into the interactions between *C. albicans* and hosts’ innate immunity during infections caused by Cas5. They could potentially lead to novel therapeutic targets aimed at disrupting these intricate pathways leading to disease progression.

### 3.5. Cas5 Is Required for C. albicans Pathogenicity in Mouse Model of OPC

**Figure 6:**
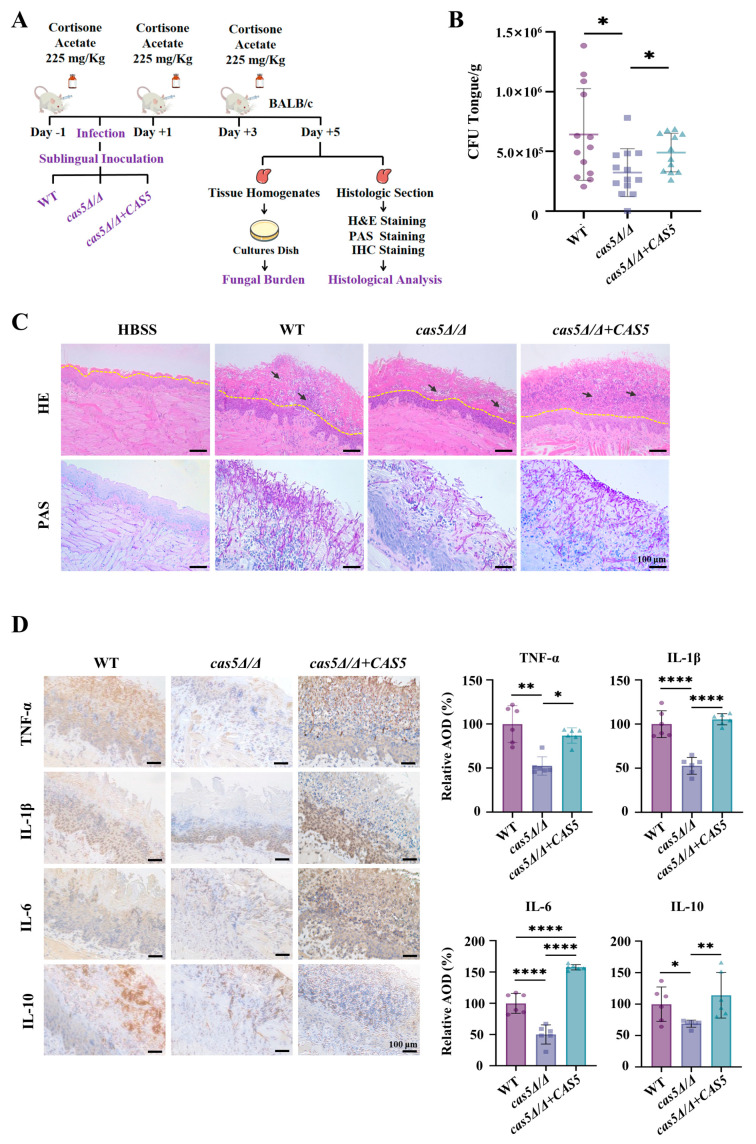
Cas5 is required for *C. albicans* pathogenicity in oropharyngeal candidiasis (OPC). (**A**). Schematic diagram on overall design evaluating effect of Cas5 on pathogenicity of *C. albicans* in OPC mouse model. (**B**). Tongue fungal burden assays. Colony Forming Units (CFU) per gram of tongue tissues were recovered from infections with *C. albicans* strains after 5 days and analyzed using Student’s *t* test (WT VS. *cas5Δ/Δ*, WT VS. *cas5Δ/Δ* + *CAS5*, and *cas5Δ/Δ* VS. *cas5Δ/Δ* + *CAS5*); Error bars represent S.D. from mean of 13 samples (one mouse in *cas5Δ/Δ* + *CAS5* infection group died from anesthesia). (**C**). Histopathological examination of mice tongues infected with *C. albicans* strains. H&E staining to detect histopathology of mice tongues harvested after 5 days of infection (**above**); PAS staining to detect colonization and invasion of mice tongues by *C. albicans* (**below**). Periostracum is area above yellow dotted line. Inflammatory cell infiltrations are indicated by black arrows. Sterile HBSS is used as control. Bar: 100 μm. (**D**). IHC examination (**left**) and quantitative analysis (**right**) of inflammatory cytokines secretions in three mice tongues from each group after 5 days of infection (pro-inflammatory cytokines: TNF-α, IL-1β, and IL-6; anti-inflammatory cytokine: IL-10). One representative area is shown for each group. Sterile HBSS is used as control. Bar: 100 μm. Measurements were analyzed using one-way ANOVA (WT VS. *cas5Δ/Δ*, WT VS. *cas5Δ/Δ* + *CAS5*, and *cas5Δ/Δ* VS. *cas5Δ/Δ* + *CAS5*); asterisks show statistically significant differences (*, *p* < 0.05, **, *p* < 0.01; ****, *p* < 0.0001).

Owing to the deficiency of extensive virulence factors in *cas5Δ/Δ*, we evaluated the effect of Cas5 on the colonization and invasion of *C. albicans* in cortisone-immunosuppressed mice with OPC (Figure S7A). The model was established according to previous descriptions of immune cell recruitment assays. The mice were sacrificed on the fifth day post-infection, and their tongues were excised for fungal burden and histopathological examination (Figure 6A and Figure S7A). The fungal burden results showed that the mean number of fungal colonies on the tongue of mice infected with *cas5Δ/Δ* was significantly lower than that on the tongue of the control strain-infected groups (Figure 6B). Representative H&E-stained images (Figure 6C) showed that the tongue tissue infected with *cas5Δ/Δ* exhibited slight mucosal damage, a normal organizational structure in the periostracum, and infiltration of only a few inflammatory cells, similar to those in the HBSS control group. However, the structures of the tongue mucosa infected with either WT or *cas5Δ/Δ + CAS5* were severely damaged by severe inflammation. Representative PAS-stained images (Figure 6C and Figure S7C) revealed the morphology and distribution of these three strains on the mouse tongues. The tongues of mice infected with WT or *cas5Δ/Δ + CAS* formed abundant tissue-penetrating hypha, whereas the tongues of mice infected with *cas5Δ/Δ* displayed a small number of yeast-forming cells and pseudohypha-forming cells located in the periostracum of the lingual mucosa.

*C. albicans* colonization in the tongue of mice caused an inflammatory response, and oral epithelial cells released a large number of inflammatory factors, including TNF-α, IL-1β, and IL-6 [48]; additionally, overwhelming microbial inoculation causes the immune system to mount a T helper type 2 lymphocyte response that is accompanied by the secretion of the anti-inflammatory cytokine IL-10, resulting in immunosuppression [49,50]. Therefore, we detected the expression levels of cytokines in the tongues of mice infected with *C. albicans* strains using IHC staining to assess inflammation levels on the fifth day post-infection (Figure 6D). We observed that *cas5Δ/Δ* resulted in a significantly reduced secretion of TNF-α, IL-1β, IL-6, and IL-10 compared to WT and *cas5Δ/Δ + CAS5,* suggesting a reduced host inflammatory response in the absence of Cas5. This finding is consistent with our observations in both the H&E- and PAS-stained images, which showed that *cas5Δ/Δ* colonized the mouse tongue to a lesser extent. In addition, the reduced secretion of IL-10 also indicated that the depletion of Cas5 was less likely to induce host immune tolerance, which further confirmed the virulence deficiency of the *cas5Δ/Δ* strains.

## 4. Discussion

The cell wall is a highly remodeled organelle that is closely related to the survival and virulence of *C. albicans* [51,52]. As a transcription factor for cell wall integrity, Cas5 is activated under the conditions of cell wall perturbation, such as when stimulated by caspofungin, to regulate cell wall remodeling [53]. Additionally, previous studies have found that knocking out Cas5 increases the survival of *Toll* mutant flies, *Caenorhabditis elegans*, and BALB/c mice in disseminated candidiasis [27,28], and also demonstrated that Cas5 is closely associated with the development of resistance in *C. albicans* [23]. Furthermore, it has been reported that Cas5 promotes the adhesion of *C. albicans* to abiotic surfaces [29]. However, there has been no systematic elucidation of the key steps in which Cas5 participates during cell wall remodeling, or whether it participates in the interactions between *C. albicans* and the host through regulating other phenotypes in addition to morphological transition.

In this study, we found that after knocking out Cas5, the inner layer of the *C. albicans* cell wall significantly thickened, the exposure of inner-layer glycans’ β-glucan increased, the CSH decreased, and the expression of virulence-related cell wall proteins was downregulated (Figure 1A,B, Figure 2C and Figure 4E). These findings deepen our understanding of Cas5’s role in regulating cell wall integrity. Additionally, through a co-culture model of the *C. albicans* and host cells, we discovered that Cas5 also affects the adhesion of *C. albicans* to biological surfaces (Figure 3, Figures S4 and S5). The main factor influencing the adhesion of *C. albicans* to abiotic surfaces is CSH, while the factors affecting the adhesion to biological surfaces include not only CSH but also adhesion proteins in the cell wall [54,55]. In our study, we demonstrated that the expression levels of the key adhesion proteins, Als1 and Csp37, in the cell wall were downregulated in *C. albicans* lacking Cas5 compared to the WT strain (Figure 2C). This finding aligns with a previous report indicating that Cas5 cooperates with Efg1 by co-binding to the *ALS1* promoter to enhance its expression [24]. Additionally, we found that the absence of Cas5 resulted in the reduced expression of the Cat1 and increased expression of Hsp90 (Figure 2C), which promotes hyphal formation in *C. albicans* [41,43]. Given that hyphal formation plays a pivotal role in adhesion enhancement, Cas5 additionally governs adhesion through its regulation of the morphological transition in *C. albicans*. Furthermore, under 37 °C cultivation in the YPD medium, Cas5 depletion resulted in a significant reduction in CSH. These findings collectively suggest that Cas5 mediates cell wall remodeling through multiple pathways, ultimately influencing the adhesive capacity of *C. albicans*. Given the role of Cas5 as a transcriptional regulator, we propose that it modulates adhesion-related processes by regulating the expression of *ALS1*, *CSP37*, and *CAT1* genes, thereby affecting the production of their encoded proteins.

The establishment of infection by *C. albicans* in vivo also requires a morphological transition from yeast to hypha after adhesion, to penetrate host epithelial and endothelial barriers and form biofilms with complex structures, thereby further enhancing the interaction between *C. albicans* and the host [56]. In our study, within 24 h of observation, the Cas5 knockout strain showed a delay of hypha development and decreased hypha frequencies in a medium simulating blood-borne infection (RPMI 1640 with 10% FBS) (Figure 4A,B). The adhesion assay (Figure 3, Figures S4 and S5) also showed that the Cas5 knockout strain formed only pseudohypha even after 120 min of incubation in the RPMI 1640 medium containing 10% FBS, whereas the WT strain developed elongated hypha that intertwined. Due to the defect in the hyphal formation, the depletion of Cas5 further reduced the ability of *C. albicans* to form biofilms (Figure 4D). In the subsequently established OPC model, we also observed the impaired hyphal development of the Cas5 knockout strain in PAS images of mouse tongues (Figure 6B and Fiugre S7C). These findings are consistent with previous reports in the *Caenorhabditis elegans* disseminated infection model [27]. Notably, Cas5 was reported to enhance the promoter-binding capability of Efg1 in response to cell wall stress. Efg1 is also one of the best characterized regulators to induce hyphal morphogenesis in *C. albicans* [57]. Therefore, we speculate that Cas5 may enhance the *C. albicans* morphology transition through the interaction with Efg1. However, an in vitro study showed that *C. albicans* lacking Cas5 exhibited enhanced yeast-to-hypha transition in the YPD medium with 10% serum within 3 h of observation [56]. These different results may be due to differences in the growth conditions and parental strains used for Cas5 gene knockout, suggesting that inhibiting Cas5 in humoral and blood environments can hinder hypha development. Overall, combining the impact of Cas5 on survival rates in disseminated animal models reported in the previous literature [27,28] and our findings, we propose that Cas5 mediates key adhesion factors—CSH and cell wall adhesion proteins—and hypha formation to increase the virulence of *C. albicans* in vivo. OPC is characterized by fungal overgrowth and the invasion of superficial tissues, resulting in hyper-inflammation and immune tolerance [50]. In fact, hyper-inflammation may be a consequence of unnecessarily prolonged or exaggerated proinflammatory immune responses post-infection [58]. The key innate immune cells that are recruited to the site of infection include neutrophils and macrophages [59]. Our study found that in the OPC model, one day after infection, *C. albicans* lacking Cas5 recruited more neutrophils compared to the WT and complemented strains (Figure 5A). Generally, the cell walls of hypha-form *C. albicans* expose more β-glucan compared to the yeast-form *C. albicans*, which theoretically should stimulate more phagocyte recruitment [47]. However, the Cas5 knockout strain showed decreased hypha frequencies both in vivo and in vitro but recruited more neutrophils compared to the WT and complemented strains (Figure 4A–C, Figure 5A and Figure 6C). However, with β-glucan masking, C. *albicans* successfully colonized the tongue mucosa, which induces the secretion of the pro-inflammatory cytokines (such as IL-1β and IL-6), and chemokines form epithelial cells that are required for more immune cell recruitment [59,60]. At this point, even without direct contact with invading hypha, neutrophils respond to the cytokines and growth factors released by epithelial cells and release TNFα in turn, which subsequently triggers a protective effect in epithelial cells by upregulating TLR4 [59]. This is consistent with the results observed five days post-infection; we found that the WT strain and complemented strain induced a more intense inflammatory response (increased production of TNF-α, IL1β, and IL-6) and greater immune tolerance (increased secretion of IL-10) in tongues than in the Cas5 knockout strain, with severe tissue damage, while the *C. albicans* lacking Cas5 only had mild inflammation and tissue damage (Figure 6D). Similarly, five days after infection, PAS staining and fungal burden measurements showed that the Cas5 knockout group had fewer *C. albicans* colonies on the tongues of mice compared to the other two groups (Figure 6B,C and Figure S7C). These results link the Cas5-mediated exposure of cell wall β-glucan with the milder inflammation and lower fungal load in the tongues of the Cas5 knockout group mice during the later stages of infection. Additionally, a previous study revealed that less virulent *C. albicans* strains are more easily cleared by the host immune system [58], which is consistent with our findings. Overall, these results further reveal that the Cas5-mediated exposure of cell wall β-glucan, morphological transitions, and adhesion are involved in the colonization of *C. albicans* in vivo, deepening our understanding of how Cas5 mediates the interaction between *C. albicans* and the host.

Building on the critical role of Cas5 in maintaining cell wall integrity and pathogenicity in *C. albicans*, further investigations into its specific signaling pathways will be essential to uncover the additional developmental processes it may regulate. Moreover, the regulatory role of Cas5 in morphological transitions also highlights the potential of developing compounds targeting Cas5 or its downstream effectors, which may inhibit hyphal formation and biofilm development, thereby enhancing therapeutic efficacy against biofilm-associated infections. In summary, we believe that elucidating the mechanisms of Cas5 in *C. albicans* pathogenicity will significantly advance the development of innovative strategies for managing these infections.

## 5. Conclusions

Cas5 modulates *C. albicans* cell wall remodeling by masking cell wall β-glucan, altering cell surface hydrophobicity (CSH), and regulating the expression of cell wall proteins (CWPs). Additionally, Cas5 plays a crucial role in inhibiting neutrophil recruitment and enhancing *C. albicans* adhesion to host cells, as well as facilitating morphological transitions. These actions promote the colonization and invasion of *C. albicans* in oropharyngeal candidiasis pathogenesis. Our findings deepen the understanding of Cas5’s role in maintaining cell wall integrity and highlight the connection between Cas5-mediated cell wall remodeling and host interactions.

## Data Availability

The mass spectrometry proteomics data have been deposited to the ProteomeXchange Consortium (https://proteomecentral.proteomexchange.org, accessed on 6 May 2024) via the iProX partner repository [62,63] with the dataset identifier PXD052043 [64]. The rest of the data generated during the study are available at figshare (http://doi.org/10.6084/m9.figshare.25729341, accessed on 7 May 2024) [65].

## Supplementary Materials

The following supporting information can be downloaded at: https://www.mdpi.com/article/10.3390/microorganisms13030683/s1, Figure S1: Schematic diagram of the construction of *cas5Δ/Δ*; Figure S2: Schematic diagram of *CAS5* expression plasmid construction; Figure S3: TEM images of cell wall inner layer thickness in various *C. albicans* strains; Figure S4: The adhesion of WT, *cas5Δ/Δ* and *cas5Δ/Δ + CAS5* on EA. hy926 cells and L-929cells after 30 min incubation; Figure S5: The adhesion of WT, *cas5Δ/Δ* and *cas5Δ/Δ + CAS5* on EA. hy926 cells and L-929 cells after 90 min incubation; Figure S6: Cas5 promotes biofilm formation of *C. albicans*; Figure S7: Cas5 promotes C. albicans pathogenicity in the mouse model of oropharyngeal candidiasis; Table S1: Sequences and descriptions of the synthetic oligonucleotides used in this study to construction *CAS5* null mutant (*cas5Δ/Δ*) and *CAS5* compensation strain (*cas5Δ/Δ + CAS5*); Table S2: Descriptive statistics of cell wall inner layer thickness in various *C. albicans* strains grown in RPMI 1640 medium containing 10% FBS at 37 °C in Figure 1A(a); Table S3: Descriptive statistics of cell wall inner layer thickness in various *C. albicans* strains grown in YPD medium at 30 °C in Figure 1A(b); Table S4: Descriptive statistics of cell wall β-glucan exposures in various *C. albicans* strains grown in YPD medium at 30 °C in Figure 1B(a); Table S5: Descriptive statistics of cell wall mannan exposures in various *C. albicans* strains grown in YPD medium at 30 °C in Figure 1B(b); Table S6: Descriptive statistics of cell wall chitin exposures in various *C. albicans* strains grown in YPD medium at 30 °C in Figure 1B(c); Table S7: Descriptive statistics of cell wall β-glucan exposures in various *C. albicans* strains grown RPMI 1640 medium containing 10% FBS at 37 °C in Figure S3C; Table S8: Descriptive statistics of hyphae frequencies (%) in various *C. albicans* strains in Figure 4B; Table S9: Descriptive statistics of relative biofilm mass (%) of various *C. albicans* strains in Figure 4D; Table S10: Descriptive statistics of CSH (%) of various *C. albicans* strains grown in YPD medium at 37 °C and room temperature (25 °C) in Figure 4E; Table S11: Descriptive statistics of neutrophils recruitments in *C. albicans* strains-infected mice tongue tissues in Figure 5A; Table S12: Descriptive statistics of macrophages recruitments in *C. albicans* strains-infected mice tongue tissues in Figure 5B; Table S13: Descriptive statistics of fungal burdens of *C. albicans* strains-infected mice tongue tissues in Figure 6B; Table S14: Descriptive statistics of relative TNF-α secretion (%) in *C. albicans* strains-infected mice tongue tissues; Table S15: Descriptive statistics of relative IL-1β secretion (%) in *C. albicans* strains-infected mice tongue tissues; Table S16: Descriptive statistics of relative IL-6 secretion (%) in *C. albicans* strains-infected mice tongue tissues; Table S17: Descriptive statistics of relative IL-10 secretion (%) in *C. albicans* strains-infected mice tongue tissues. Reference [61] is cited in the Supplementary Materials.
