# Supplementary material for: Cas5 Regulates the Exposure of β-Glucan, the Cell Surface Hydrophobicity, and the Expression of Cell Wall Proteins to Remodel the Candida albicans Cell Wall and Participates in the Recruitment of Neutrophils
Source: Microorganisms. 2025 Mar 19;13(3):683. doi: 10.3390/microorganisms13030683 (PMC11944837; doi:10.3390/microorganisms13030683)
Supplement: Supplementary file 1 [file microorganisms-13-00683-s001.zip › microorganisms-3489927-supplementary.pdf]

Cas5 Regulates the Exposure of β-Glucan, the Cell Surface Hydrophobicity, and the Expression of Cell Wall Proteins to Remodel the *Candida albicans* Cell Wall and Participates in the Recruitment of Neutrophils

1 Supplementary Materials and Methods .... - 2 -

1.1 Plasmid and strain construction .... - 2 -

1.2 *CAS5* gene expression analysis .... - 2 -

2 Supplementary Tables and Figures .... - 4 -

2.1 Oligonucleotides used to construct plasmids .... - 4 -

2.2 Schematic diagram of *CAS5* null mutant construction and PCR validation .... - 6 -

2.3 Schematic diagram of *CAS5* expression plasmid construction and RT-qPCR validation .... - 8 -

2.4 The cell wall glycans remodeling of *C. albicans* .... - 10 -

2.5 Adhesion on host cells .... - 10 -

2.6 The morphology transition of *C. albicans* .... - 15 -

2.7 The pathogenicity of *C. albicans* in the mouse model of oropharyngeal candidiasis (OPC) .... - 16 -

2.8 The descriptive statistical analysis in this study .... -18 -

3 References .... - 18 -

1 Supplementary Materials and Methods

1.1 Plasmid and strain construction

CRISPR system was used to construct *CAS5* null mutant (*cas5Δ/Δ*). In details, two complementary oligos (Table S1) matched the *CAS5* gene with restriction sites were synthesized by Beijing Tsingke Biotech Co., Ltd. To obtain guide sequence of *CAS5*, the oligos were annealed at 70°C for 20 min. Then the guide sequence was ligated to the pV1093 plasmid[1] digested with *BsmB*I (New England Biolabs, NEB) by T4 ligase (New England Biolabs, NEB) at 37°C. Correct guide expression plasmids (pV1093-*CAS5*) were confirmed by sequencing. The guide expression plasmids were linearized by digesting with *Kpn1* and *Sac1* (New England Biolabs, NEB) before transformation for efficient targeting to the *ENO1* locus (Figure S1A). The repair templates were amplified with 500 bp upstream and 500 bp downstream of the open reading frame (ORF) of *CAS5* gene using primers (Figure S1B and Table S1). The PCR products of upstream and downstream were ligated by overlap PCR and purified by 1% agarose gel. To create *cas5*Δ/Δ, both the linearized pV1093-*CAS5* plasmids (10 μg) and the repair templates were transformed into WT by electrotransformation and were selected on YPD plates with nourseothricin at a concentration of 200 μg/ml (Figure S1C). The genomes of positive colonies were extracted after growing in YPD medium overnight and CRISPR-mutagenized loci were verified by sequence analysis of PCR products amplified from the target locus (Figure S1D-E). To create the *cas5*Δ/Δ+*CAS5* stain, the constructed modified pCaEXP (pCaEXP-M) plasmids containing *CAS5* ORF (The

guide sequence of *CAS5* was replaced by their synonymous codons) was transformed into *cas5Δ/Δ* and screened on the YPD containing geneticin (200 μg/ml) (Figure S2).

1.2 *CAS5* Gene expression analysis.

The various *C. albicans* strains of exponential growth phase grown in YPD medium at 30℃ were harvested by centrifugation. RNA was isolated with the Trizol (Sangon Biotech, China) and then were converted to cDNA using PrimeScript Fast RT reagent Kit (TAKARA, Japan). Subsequently, the gene expression levels in *C. albicans* were detected by quantitative real-time PCR (qPCR) using the SYBR green PCR kit (ABclonal, China) and a CFX Connect Real-Time system (Bio-rad, USA) following the manufacturer's protocol. The results were analyzed by the $2^{-\triangle\triangle CT}$ method[2] using *18S* rRNA as the endogenous control. The primers for qPCR are also given in Table S1. All the gene expression levels were determined in three biological replicates, each tested in triplicate.

2 Supplementary Tables and Figures

2.1 Oligonucleotides used to construct plasmids

Table S1. Sequences and descriptions of the synthetic oligonucleotides used in this study to construction *CAS5* null mutant (*cas5Δ/Δ*) and *CAS5* compensation strain (*cas5Δ/Δ+CAS5*)

**Primer / Oligonucleotides**	**Sequence (5'-3')**	**Description**
CAS5_sgRNA top	ATTTGGATGAGGACATTGCCAATTCAGGG	Oligomers to obtain guide sequence of *CAS5*
CAS5_sgRNA bottom	AAAACCCTGAATTGGCAATGTCCTCATCC	
CAS5 upstream donor_F	TTGATTTAATTAGTACTTGTTTCCAATTCAC	Primers to obtain repaired template (up)
CAS5 upstream donor_R	GTAAGCAATGGTGTACTAATATAGTATATGTTAAAGTCTATATTTACCAACAAAGG	
CAS5 downstream donor_F	TATAGACTTTAACATATACTATATTAGTACACCATTGCTTACCC	Primers to obtain repaired template (down)
CAS5 downstream donor_R	GGGTAAGCAATGGTGTACTAATAT	
CAS5 upstream donor+155_F	GGAGACCAGAAGAAATTTATCAAC	Primers to check *CAS5* mutation
CAS5 downstream donor+252_R	CACCTGATTGTCGGGATTG	
Linearized pCaEXP-M for CAS5_F	GATCCTCTAGAGTCGACCTGC	Primers to create linearized modified pCaEXP (pCaEXP-M) vector for ligation *CAS5*
Linearized pCaEXP-M for CAS5_R	CGGGGAGGGTATTTACTTTTAAATATAG	
CAS5-ORF-pCaEXP_F	TAAAAGTAAATACCCTCCCCGATGGAGAATTATTTATTAAGTTCGCCG	Primers to create *CAS5* insert for ligation with linearized pCaEXP vector
CAS5-ORF-pCaEXP_R	CGACTCTAGAGGATCTTAGGAAACTTCTTTGTTTTCATTCAACAC	
CAS5-F	GGCGACAGGAAATGGAAAGG	Primers to detect the *CAS5* gene expression in WT and *cas5Δ/Δ+CAS5* by qPCR
CAS5-R	CACTGTACCGTCATCGTCGT	

Primer / Oligonucleotides	Sequence (5’-3’)	Description
18S-F	AATTACCCAATCCCGACAC	Primers of reference gene to calibrate target genes expression in WT and *cas5Δ/Δ+CAS5* by qPCR
18S-R	TGCAACAACTTTAATATACGC	

2.2 Schematic diagram of *CAS5* null mutant construction and PCR validation

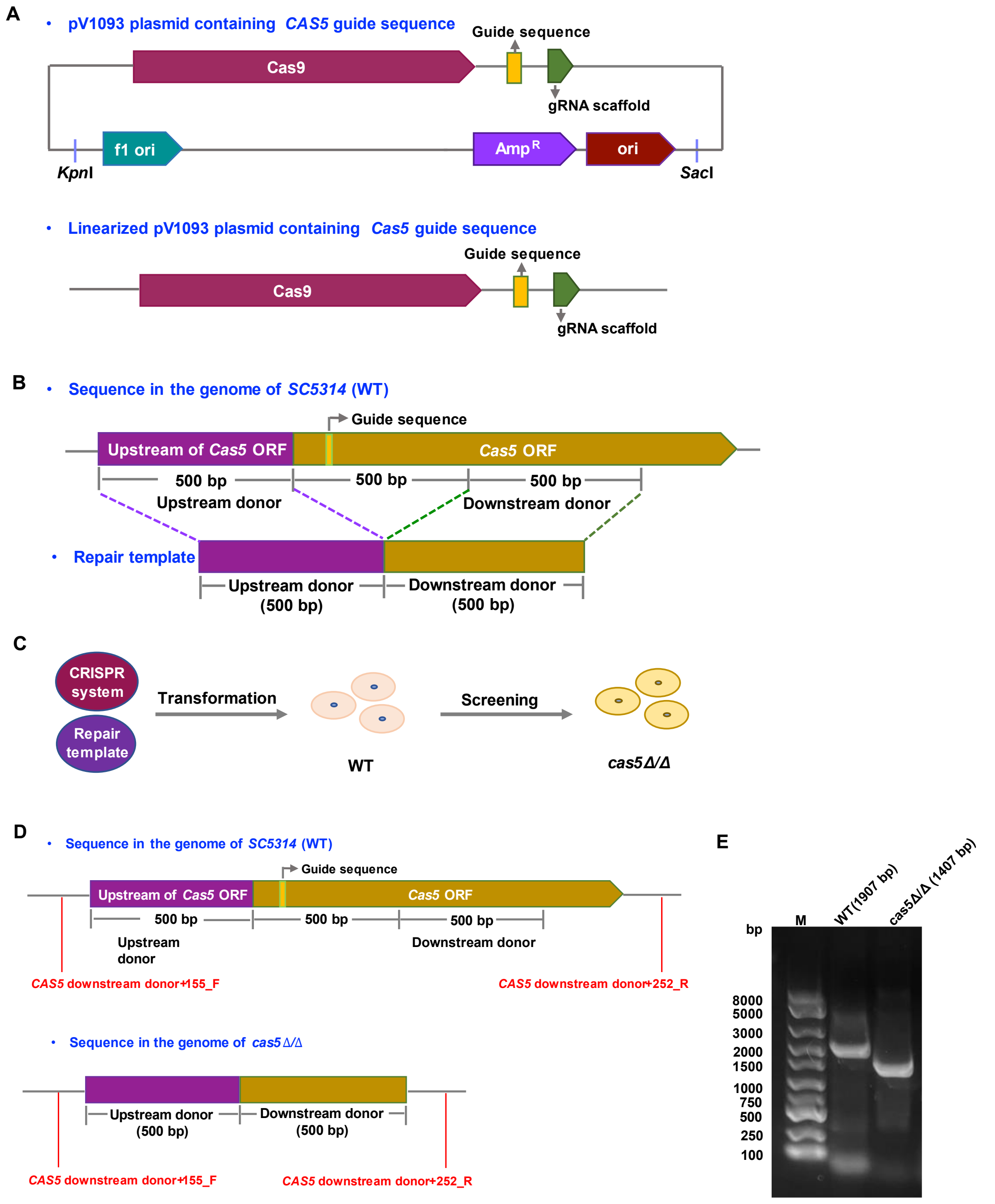

Figure S1 Schematic diagram of the construction of *cas5Δ/Δ*. A. Schematic diagram of CRISPR system containing the guide sequence to target *CAS5* gene, **B.** Design schematic of repair template, **C.** Construction schematic of *CAS5* null mutant, **D.** Sequence schematic of *CAS5* locus in *cas5Δ/Δ*, **E.** DNA gel of PCR products amplified

by primers. M: DNA marker, PCR product (1907 bp) amplified by primers (*CAS5* upstream donor+155_F and *CAS5* downstream donor+252_R) from genome of WT, 2: PCR product (1407 bp) amplified by primers (*CAS5* upstream donor+155_F and *CAS5* downstream donor+252_R) from genome of *cas5Δ/Δ*.

2.3 Schematic diagram of *CAS5* expression plasmid construction and RT-qPCR validation

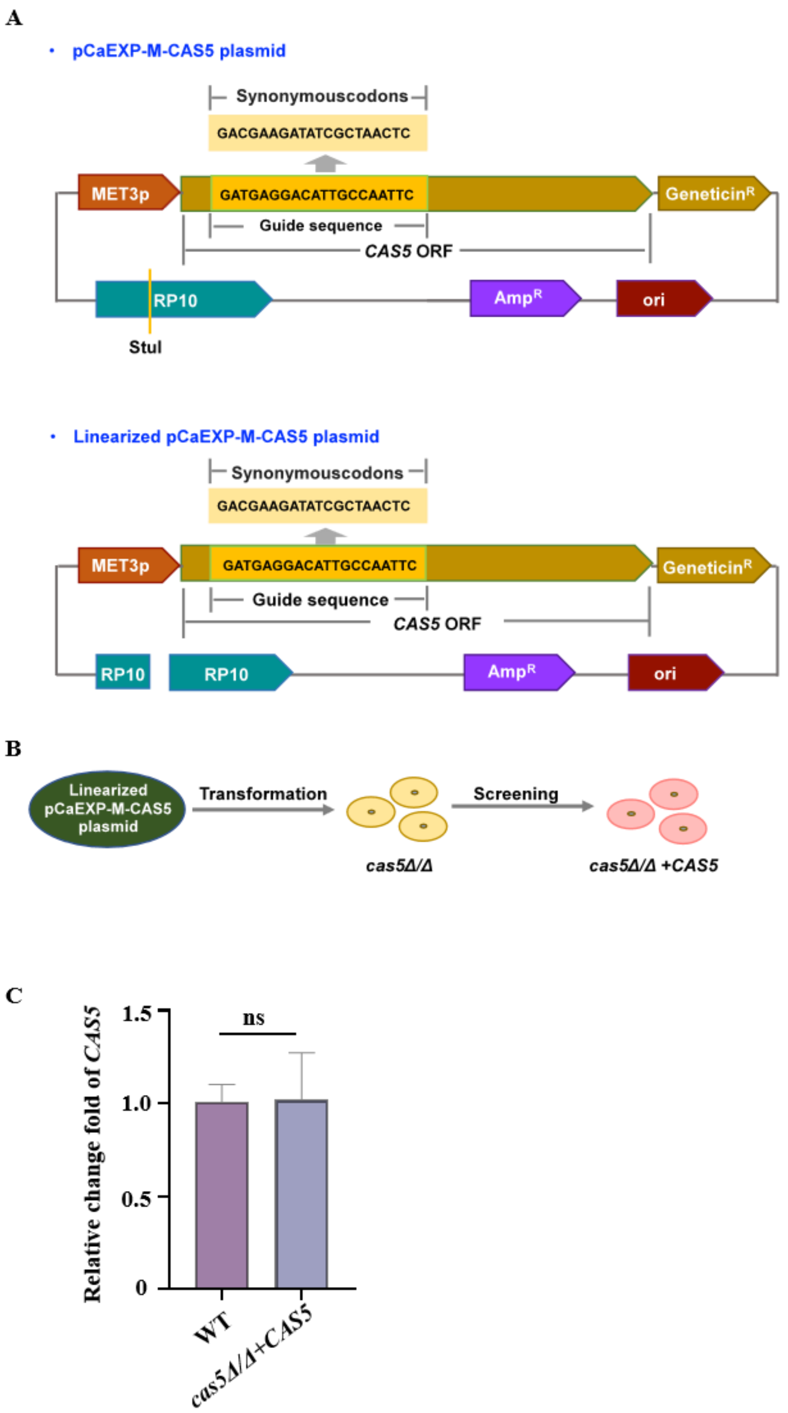

Figure S2 Schematic diagram of *CAS5* expression plasmid construction. A. Schematic construction of *CAS5* expression plasmid (pCaEXP-M-CAS5) and *CAS5*

gene expression cassette (Linearized pCaEXP-M-CAS5) that the pCaEXP-M-CAS5 is linearized at RP10 locus by digestion with *Stu*I. **B**. Construction schematic of *CAS5* complemented strain. **C.** Relative expression levels of *CAS5* gene in WT and *cas5*Δ/Δ+*CAS5*, detected by RT-qPCR (primers presented in Table S1), ns: no statistical difference.

2.4 The cell wall glycans remodeling of *C. albicans*

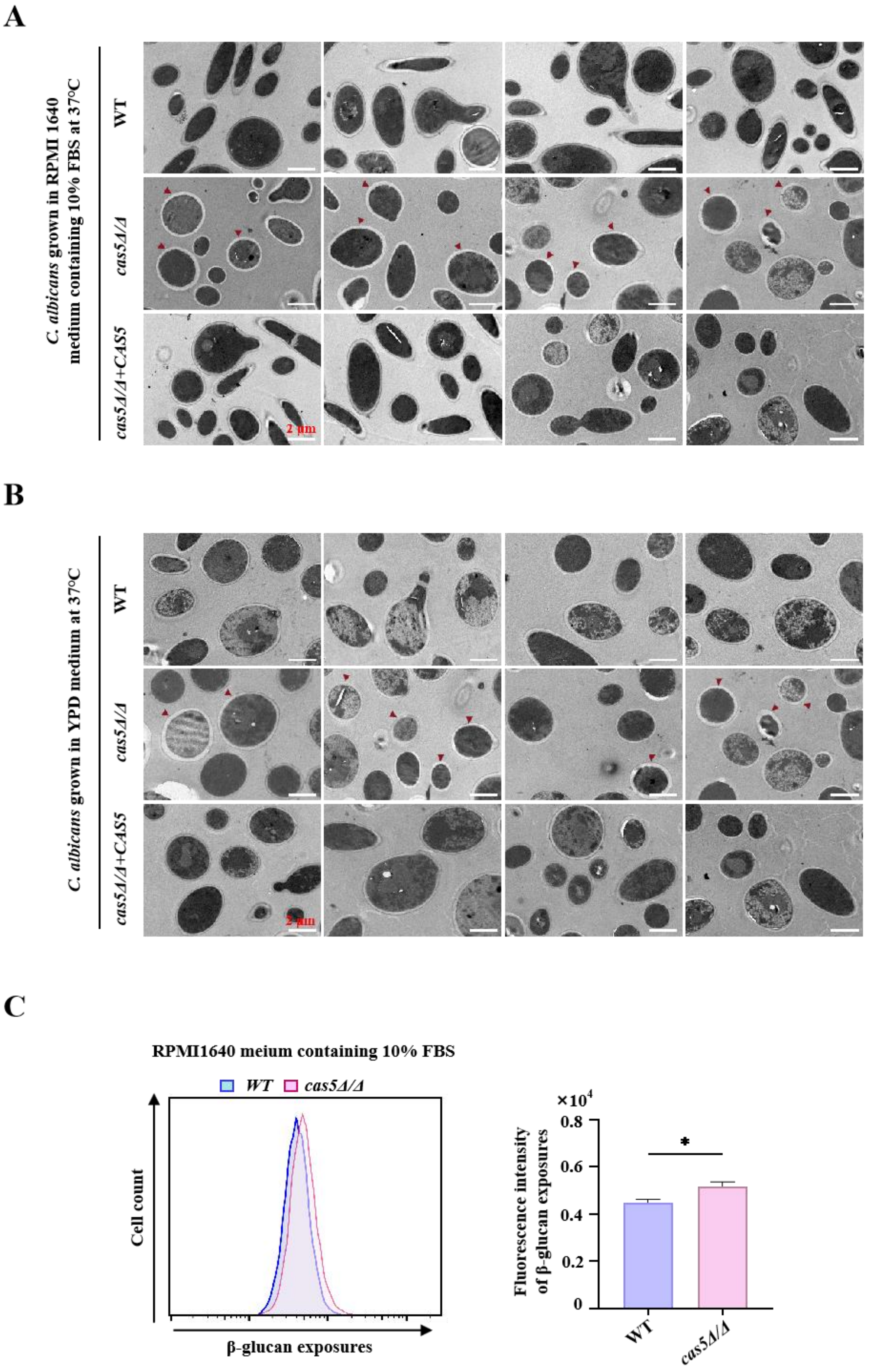

Figure S3 TEM images of cell wall inner layer thickness in various *C. albicans* strains. A. *C. albicans* grown in YPD medium at 30°C overnight and subsequently cultured in RPMI1640 medium containing 10% FBS at 37°C for 90 min. **B.** *C. albicans* grown in YPD medium at 30°C overnight and subsequently cultured in YPD medium at 30°C for 90 min. *cas5Δ/Δ* cells with a thickened cell wall inner layer are indicated by red arrows. Bar: 2 μm. C. Flow cytometry analysis (left) of the cell wall β-glucan exposures in various *C. albicans* strains grown in YPD medium at 30°C overnight and subsequently cultured in RPMI1640 medium containing 10% FBS at 37°C for 90 min.. Histogram plots (right) are representative of data collected in three independent replicate experiments, with error bars; Measurements were analyzed using the student's T test (WT VS. *cas5Δ/Δ*); Asterisks show statistically significant differences (*, $p <$ 0.05).

2.5 Adhesion on host cells

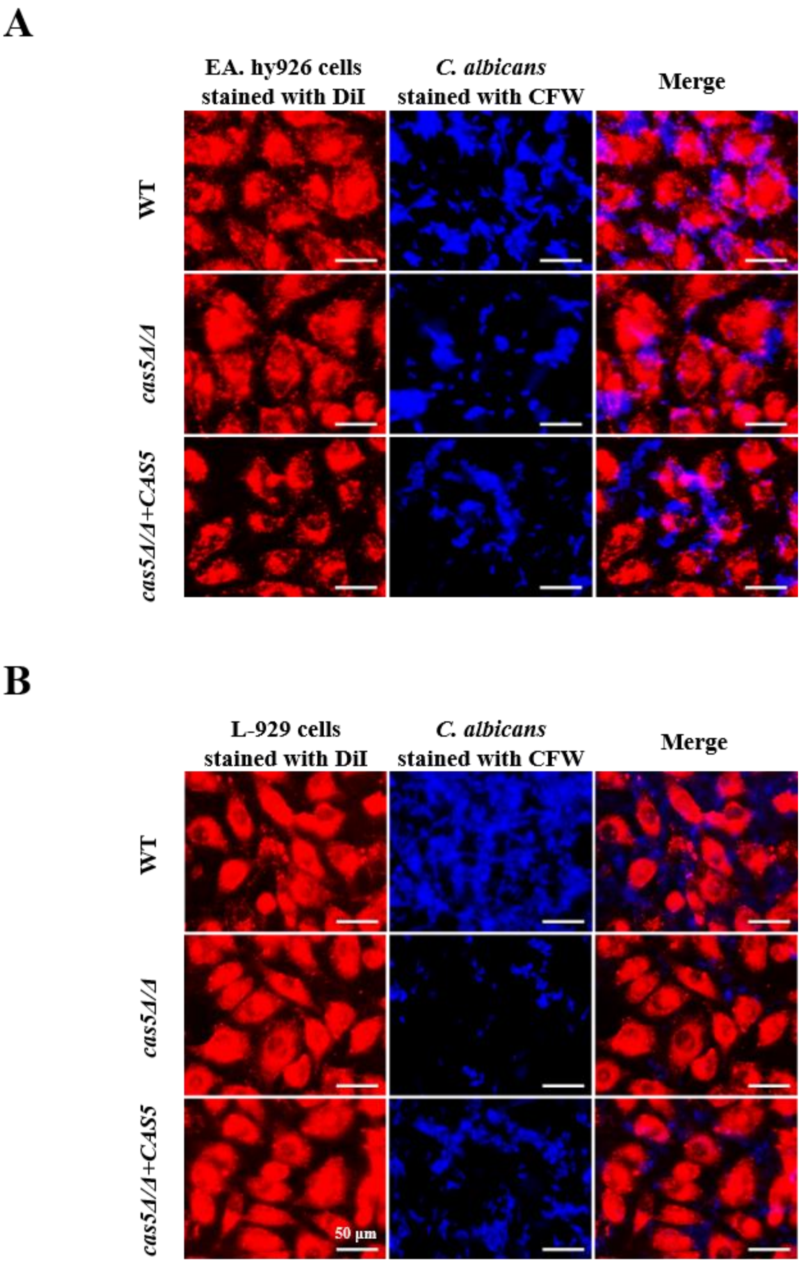

Figure S4 The adhesion of WT, *cas5Δ/Δ* and *cas5Δ/Δ+CAS5* on EA. hy926 cells and L-929cells after 30 min incubation. A. Fluorescence micrographs of the adhesion ability of *C. albicans* strains (exponential growth phase) to EA. hy926 cells in RPMI1640 medium at 37°C. **B**. Fluorescence micrographs of the adhesion ability of *C.*

albicans strains to L-929 cells incubated in RPMI1640 medium at 37°C. Calcofluor white (CFW): staining *C. albicans*, DiI: staining EA. hy926 / L-929 cells. Bar: 50 μm.

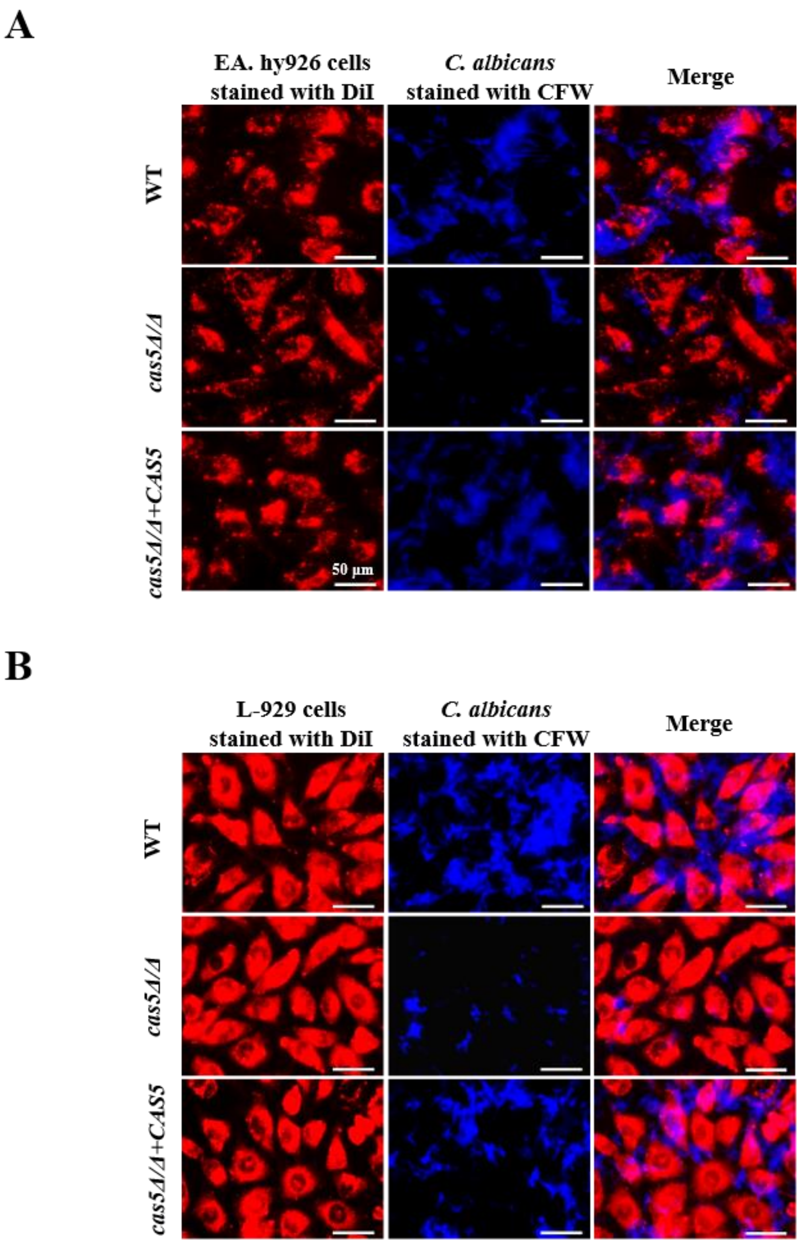

Figure S5 The adhesion of WT, *cas5Δ/Δ* and *cas5Δ/Δ+CAS5* on EA. hy926 cells and L-929cells after 90 min incubation. A. Fluorescence micrographs of the adhesion ability of *C. albicans* strains (exponential growth phase) to EA. hy926 cells incubated in RPMI1640 medium at 37°C. **B**. Fluorescence micrographs of the adhesion ability of

C. albicans strains to L-929 cells incubated in RPMI1640 medium at 37℃. Calcofluor white (CFW): staining *C. albicans*, DiI: staining EA. hy926 / L-929 cells. Bar: 50 μm.

2.6 The morphology transition of *C. albicans*

A

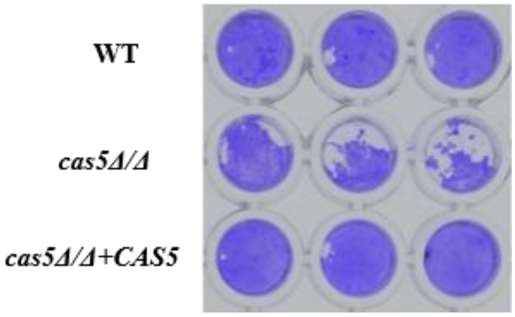

Figure S6 Cas5 promotes biofilm formation of *C. albicans*. A. Images of the biofilms formed by various *C. albicans* strains in 96-well plates after incubation in RPMI 1640 medium supplemented with 10% FBS at 37°C for 48 h. The biofilms were stained with crystal violet.

2.7 The pathogenicity of *C. albicans* in the mouse model of oropharyngeal candidiasis (OPC)

A

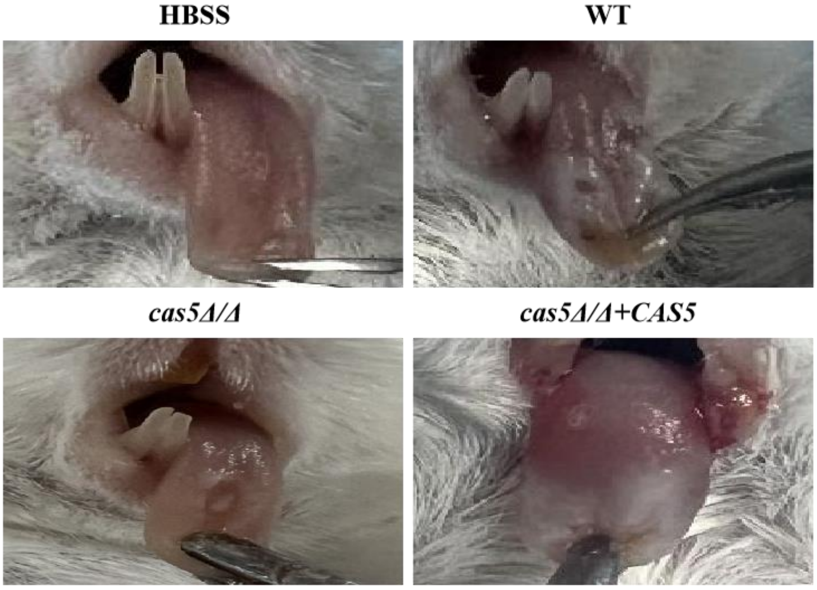

B

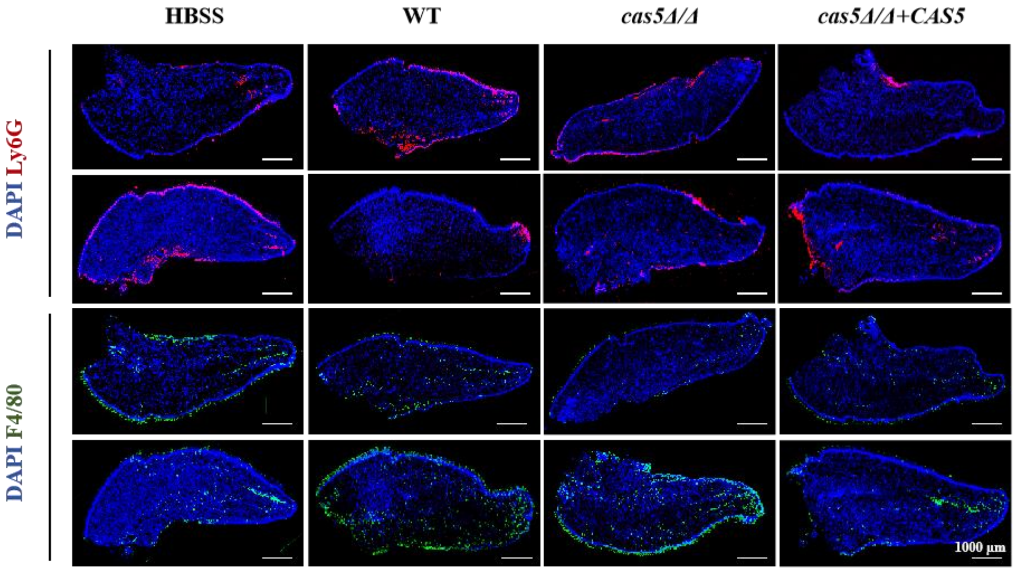

C

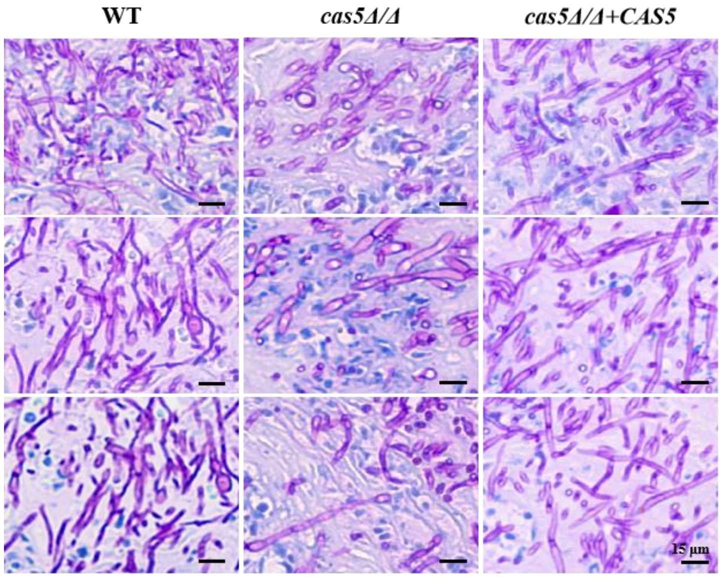

Figure S7 Cas5 promotes *C. albicans* pathogenicity in the mouse model of oropharyngeal candidiasis. A. Representative images of *C. albicans* strains-infected mice tongue tissues after 5 days of infection. **B**. Fluorescence micrographs of the neutrophils and macrophages recruitments in *C. albicans* strains-infected mice tongue tissues. DAPI: staining DNA to localize cells, rabbit anti-ly6G antibody: neutrophils labeling, rabbit anti-F4/80 antibody: macrophages labeling. Two representative samples are shown for each group. The sterile HBSS is used as the control. Bar: 1000 μm. C. Representative magnification areas of PAS staining to detect the morphology transition from yeast to hyphae by *C. albicans* on mice tongues. Bar: 15 μm.

2.8 The descriptive statistical analysis in this study

2.8.1 Descriptive statistics of *C. albicans* cell wall inner layer thickness in TEM images (Figure1)

Table S2. Descriptive statistics of cell wall inner layer thickness in various *C. albicans* strains grown in RPMI 1640 medium containing 10% FBS at 37 ℃ in Figure 1A(a)

**Statistic**	**WT**	***cas5Δ/Δ***	***cas5Δ/Δ+CAS5***
Sample Size (n)	18	18	18
Mean (μm)	0.1689	0.3117	0.1478
SD (μm)	0.04813	0.07801	0.03964
Median (μm)	0.1800	0.2800	0.1350
Minimum (μm)	0.08000	0.2200	0.09000
Maximum (μm)	0.2300	0.4900	0.2100
First Quartile (μm)	0.1200	0.2725	0.1200
Third Quartile (μm)	0.2100	0.3400	0.1900

Table S3. Descriptive statistics of cell wall inner layer thickness in various *C. albicans* strains grown in YPD medium at 30 ℃ in Figure 1A(b)

**Statistic**	**WT**	***cas5Δ/Δ***	***cas5Δ/Δ+CAS5***
Sample Size (n)	18	18	18
Mean (μm)	0.2089	0.2772	0.1739
SD (μm)	0.04702	0.08028	0.03415
Median (μm)	0.2100	0.2700	0.1700
Minimum (μm)	0.1200	0.1500	0.1100
Maximum (μm)	0.3200	0.4600	0.2200
First Quartile (μm)	0.1825	0.2250	0.1550
Third Quartile (μm)	0.2325	0.3300	0.2100

2.8.2 Descriptive statistics of *C. albicans* cell wall glycans exposures in flow cytometry analysis (Figure 2 and Figure S3C)

Table S4. Descriptive statistics of cell wall β-glucan exposures in various *C. albicans* strains grown in YPD medium at 30 °C in Figure 1B(a)

Statistic	WT	*cas5Δ/Δ*	*cas5Δ/Δ+CAS5*
Sample Size (n)	3	3	3
Mean	5282	6443	5280
SD	683.1	201.2	161.6
Median	5668	6454	5360
Minimum	4493	6237	5094
Maximum	5684	6639	5386

Table S5. Descriptive statistics of cell wall mannan exposures in various *C. albicans* strains grown in YPD medium at 30 °C in Figure 1B(b)

Statistic	WT	*cas5Δ/Δ*	*cas5Δ/Δ+CAS5*
Sample Size (n)	3	3	3
Mean	96770	94100	98330
SD	32650	33470	34540
Median	83300	81200	79300
Minimum	73000	69000	77500
Maximum	134000	132100	138200

Table S6. Descriptive statistics of cell wall chitin exposures in various *C. albicans* strains grown in YPD medium at 30 °C in Figure 1B(c)

Statistic	WT	*cas5Δ/Δ*	*cas5Δ/Δ+CAS5*
Sample Size (n)	3	3	3
Mean	60630	62600	61100
SD	10640	2100	9850
Median	59000	63500	61200
Minimum	50900	60200	51200
Maximum	72000	64100	70900

Table S7. Descriptive statistics of cell wall β-glucan exposures in various *C. albicans* strains grown RPMI 1640 medium containing 10% FBS at 37 ℃ in Figure S3C

**Statistic**	**WT**	***cas5Δ/Δ***	***cas5Δ/Δ+CAS5***
Sample Size (n)	3	3	3
Mean	4490	5179	4695
SD	136.4	181.4	386.4
Median	4478	5222	4561
Minimum	4360	4980	4394
Maximum	4632	5335	5131

2.8.3 Descriptive statistics of morphology transition in *C. albicans* strains (Figure4B, 4D)

Table S8. Descriptive statistics of hyphae frequencies (%) in various *C. albicans* strains in Figure 4B

**Tine (h)**	**Statistic**	**WT**	***cas5Δ/Δ***	***cas5Δ/Δ+CAS5***
0	Sample Size (n)	0	0	0
	Mean	0.000	0.000	0.000
	SD	0.000	0.000	0.000
	Median	0.000	0.000	0.000
	Minimum	0.000	0.000	0.000
	Maximum	0.000	0.000	0.000
2	Sample Size (n)	3	3	3
	Mean	96.27	33.09	97.22
	SD	4.235	8.246	4.811
	Median	97.14	31.25	100.0
	Minimum	91.67	25.93	91.67
	Maximum	100.0	42.11	100.0
5	Sample Size (n)	3	3	3
	Mean	100.0	22.29	100.0
	SD	0.000	13.64	0.000
	Median	100.0	25.00	100.0
	Minimum	100.0	7.500	100.0
	Maximum	100.0	34.38	100.0
7	Sample Size (n)	3	3	3
	Mean	100.0	25.70	100.0
	SD	0.000	15.77	0.000
	Median	100.0	29.63	100.0

Tine (h)	Statistic	WT	*cas5Δ/Δ*	*cas5Δ/Δ+CAS5*
	Minimum	100.0	8.333	100.0
	Maximum	100.0	39.13	100.0
24 h	Sample Size (n)	3	3	3
	Mean	100.0	24.64	100.0
	SD	0.000	3.705	0.000
	Median	100.0	24.14	100.0
	Minimum	100.0	21.21	100.0
	Maximum	100.0	28.57	100.0

Table S9. Descriptive statistics of relative biofilm mass (%) of various *C. albicans* strains in Figure 4D

Statistic	WT	*cas5Δ/Δ*	*cas5Δ/Δ+CAS5*
Sample Size (n)	3	3	3
Mean	100.0	63.25	100.5
SD	7.399	20.88	9.217
Median	102.7	61.86	96.06
Minimum	91.63	43.11	94.41
Maximum	105.7	84.80	111.1

2.8.4 Descriptive statistics of morphology transition in *C. albicans* strains (Figure 4E)

Table S10. Descriptive statistics of CSH (%) of various *C. albicans* strains grown in YPD medium at 37℃ and room temperature (25℃) in Figure 4E

Temperature (℃)	Statistic	WT	*cas5Δ/Δ*	*cas5Δ/Δ+CAS5*
37	Sample Size (n)	3	3	3
	Mean	22.71	14.33	23.61
	SD	3.852	3.909	3.310
	Median	20.50	12.74	22.52
	Minimum	20.48	11.46	20.99
	Maximum	27.16	18.78	27.33
25	Sample Size (n)	3	3	3
	Mean	21.44	22.17	21.52
	SD	3.612	3.685	2.498
	Median	19.55	22.65	22.05
	Minimum	19.18	18.27	18.80
	Maximum	25.61	25.60	23.72

2.8.5 Descriptive statistics of quantitive analysis of neutrophils and macrophages recruitments in *C. albicans* strains-infected mice tongue tissues (Figure 5A, B)

Table S11. Descriptive statistics of neutrophils recruitments in *C. albicans* strains-infected mice tongue tissues in Figure 5A

Statistic	WT	*cas5Δ/Δ*	*cas5Δ/Δ+CAS5*
Sample Size (n)	3	3	3
Mean	0.09419	1.881	0.09249
SD	0.07775	0.6962	0.04288
Median	0.08004	1.656	0.08474
Minimum	0.02449	1.325	0.05401
Maximum	0.1780	2.662	0.1387

Table S12. Descriptive statistics of macrophages recruitments in *C. albicans* strains-infected mice tongue tissues in Figure 5B

Statistic	WT	*cas5Δ/Δ*	*cas5Δ/Δ+CAS5*
Sample Size (n)	3	3	3
Mean	9.856	5.698	7.984
SD	1.063	3.696	5.048
Median	10.39	4.297	7.066
Minimum	8.631	2.908	3.458
Maximum	10.54	9.890	13.43

2.8.6 Descriptive statistics of fungal burdens of *C. albicans* strains-infected mice tongue tissues (Figure 6B)

Table S13. Descriptive statistics of fungal burdens of *C. albicans* strains-infected mice tongue tissues in Figure 6B

Statistic	WT	*cas5Δ/Δ*	*cas5Δ/Δ+CAS5*
Sample Size (n)	13	13	12
Mean	642277	322639	490699
SD	383886	200037	160949
Median	534704	265808	479296
Minimum	205056	1824	262500
Maximum	1384615	780952	687356

2.8.7 Descriptive statistics of inflammatory cytokines secretion quantitive analysis in *C. albicans* strains-infected mice tongue tissues (Figure 6D)

Table S14. Descriptive statistics of relative TNF-α secretion (%) in *C. albicans* strains-infected mice tongue tissues

Statistic	WT	*cas5Δ/Δ*	*cas5Δ/Δ+CAS5*
Sample Size (n)	6	6	6
Mean	100.0	52.41	87.00
SD	20.84	10.42	8.643
Median	104.1	48.56	89.73
Minimum	73.55	45.65	70.76
Maximum	121.9	73.34	93.73

Table S15. Descriptive statistics of relative IL-1β secretion (%) in *C. albicans* strains-infected mice tongue tissues

Statistic	WT	*cas5Δ/Δ*	*cas5Δ/Δ+CAS5*
Sample Size (n)	6	6	6
Mean	100.0	52.72	105.5
SD	15.20	9.527	6.333
Median	95.00	55.18	106.5
Minimum	86.62	38.08	95.80
Maximum	124.1	65.06	112.5

Table S16. Descriptive statistics of relative IL-6 secretion (%) in *C. albicans* strains-infected mice tongue tissues

**Statistic**	**WT**	***cas5Δ/Δ***	***cas5Δ/Δ+CAS5***
Sample Size (n)	6	6	6
Mean	100.0	50.10	157.7
SD	15.87	15.35	4.282
Median	101.2	51.91	157.2
Minimum	81.94	22.32	151.8
Maximum	117.1	67.22	164.4

Table S17. Descriptive statistics of relative IL-10 secretion (%) in *C. albicans* strains-infected mice tongue tissues

**Statistic**	**WT**	***cas5Δ/Δ***	***cas5Δ/Δ+CAS5***
Sample Size (n)	6	6	6
Mean	100.0	68.99	114.2
SD	27.43	5.665	36.22
Median	104.1	70.41	100.6
Minimum	64.01	57.80	80.33
Maximum	137.0	73.65	166.2

References

[1] Vyas VK, Barrasa MI, Fink GR. A Candida albicans CRISPR system permits genetic engineering of essential genes and gene families. Sci Adv. 2015;1(3). doi:ARTN e150024810.1126/sciadv.1500248.

[2] Arocho A, Chen BY, Ladanyi M, Pan QL. Validation of the 2(-Delta Delta Ct) calculation as an alternate method of data analysis for quantitative PCR of BCR-ABL P210 transcripts. Diagn Mol Pathol. 2006;15(1):56-61. doi: 10.1097/00019606-200603000-00009.
